# Evaluating the Cognitive Impacts of Drospirenone, a Spironolactone-Derived Progestin, Independently and in Combination With Ethinyl Estradiol in Ovariectomized Adult Rats

**DOI:** 10.3389/fnins.2022.885321

**Published:** 2022-05-25

**Authors:** Stephanie V. Koebele, Mallori L. Poisson, Justin M. Palmer, Claire Berns-Leone, Steven N. Northup-Smith, Veronica L. Peña, Isabel M. Strouse, Haidyn L. Bulen, Shruti Patel, Corissa Croft, Heather A. Bimonte-Nelson

**Affiliations:** ^1^Department of Psychology, Arizona State University, Tempe, AZ, United States; ^2^Arizona Alzheimer’s Consortium, Phoenix, AZ, United States

**Keywords:** drospirenone (DRSP), ethinyl estradiol (EE), cognition, rat model, contraceptive

## Abstract

Oral contraceptives and hormone therapies require a progestogen component to prevent ovulation, curtail uterine hyperplasia, and reduce gynecological cancer risk. Diverse classes of synthetic progestogens, called progestins, are used as natural progesterone alternatives due to progesterone’s low oral bioavailability. Progesterone and several synthetic analogs can negatively impact cognition and reverse some neuroprotective estrogen effects. Here, we investigate drospirenone, a spironolactone-derived progestin, which has unique pharmacological properties compared to other clinically-available progestins and natural progesterone, for its impact on spatial memory, anxiety-like behavior, and brain regions crucial to these cognitive tasks. Experiment 1 assessed three drospirenone doses in young adult, ovariectomized rats, and found that a moderate drospirenone dose benefited spatial memory. Experiment 2 investigated this moderate drospirenone dose with and without concomitant ethinyl estradiol (EE) treatment, the most common synthetic estrogen in oral contraceptives. Results demonstrate that the addition of EE to drospirenone administration reversed the beneficial working memory effects of drospirenone. The hippocampus, entorhinal cortex, and perirhinal cortex were then probed for proteins known to elicit estrogen- and progestin- mediated effects on learning and memory, including glutamate decarboxylase (GAD)65, GAD67, and insulin-like growth factor receptor protein expression, using western blot. EE increased GAD expression in the perirhinal cortex. Taken together, results underscore the necessity to consider the distinct cognitive and neural impacts of clinically-available synthetic estrogen and progesterone analogs, and why they produce unique cognitive profiles when administered together compared to those observed when each hormone is administered separately.

## Introduction

Most individuals use some form of contraception during their reproductive years (Daniels and Abma, 2018). In the past several decades, there has been a rise in the popularity of exogenous hormone-containing methods, including oral contraceptives, intrauterine devices, vaginal rings, and subcutaneous implants due to their high reliability not only in preventing unintended pregnancy, but also for their value in treating a range of other health-related indications such as endometriosis, acne, and premenstrual dysphoric disorder (PMDD; Dayal and Barnhart, 2001). It is currently estimated that 79.3% of women have used the oral contraceptive pill at some point during their life; 13.9% of United States women ages 15–44 report current use of the pill between 2015 and 2017; this percentage increases to 19.5% for users in the 20–29 year old age range (Centers for Disease Control and Prevention, 2019). Likewise, some women undergoing the menopause transition opt to take hormone therapy to alleviate unwanted symptoms including hot flashes, dyspareunia, and vaginal dryness (Pinkerton et al., 2017). Furthermore, oral contraceptives are often prescribed for pregnancy prevention during the menopause transition when fertility is variable (Ikhena and Johnson, 2012; Liu, 2021). Thus, a significant number of people will have had exposure to hormone-containing therapies at some point in the reproductive lifespan, and it is imperative to understand the long-term health effects of hormone-containing contraceptives and menopausal hormone therapies.

Progestins act by inhibiting ovulation and altering the uterine and cervical environment for pregnancy prevention (Rivera et al., 1999). If an estrogen-containing formulation is used and the uterus is intact, the progestin component also facilitates prevention of endometrial hyperplasia, making combined oral contraceptives the most popular form of oral contraceptive use (Hall and Trussell, 2012). Due to the low oral bioavailability of natural 17β-estradiol (E2) and progesterone, synthetic forms of estrogen and progesterone are most often utilized. Ethinyl estradiol (EE) is the synthetic estrogen used in nearly all combined oral contraceptive formulations. However, a wide range of progesterone synthetics exist, collectively called progestins. Progestins are derived from a variety of parent molecules structurally related to either testosterone or progesterone. This results in different pharmacological and pharmacokinetic profiles, including variable affinities to the steroid hormone receptors for progesterone, androgens, estrogens, glucocorticoids, and mineralocorticoids (Schindler et al., 2003; Kuhl, 2005; Sitruk-Ware and Nath, 2010). Although progestins have similar progesterone-like effects peripherally, research suggests that many progestins have a negative impact on cognition and reverse neuroprotective estrogen effects (Chesler and Juraska, 2000; Bimonte-Nelson et al., 2004b,^2006^; Rosario et al., 2006; Harburger et al., 2007, 2009; Braden et al., 2010, 2011, 2015; Lowry et al., 2010), while others have neutral or even beneficial effects when administered independently (Braden et al., 2017; Prakapenka et al., 2018). Given the prevalence and diversity of progestins used in contraceptives and hormone therapies, it is of critical importance to better understand how synthetic hormones impact the brain and behavior beyond their prescribed uses.

While most United States Food and Drug Administration (FDA)-approved progestins are structurally similar to testosterone and progesterone, the progestin drospirenone is derived from a novel source: spironolactone, an anti-androgenic aldosterone antagonist (Krattenmacher, 2000; Archer et al., 2015). This makes the molecular structure and function of drospirenone unique. Aldosterone is an adrenal-derived hormone that regulates water retention and blood pressure; thus, beyond drospirenone’s capacity to bind to the progesterone receptor with high affinity and its structural similarity to progesterone compared to other clinically-available progestins (Muhn et al., 1995; Fuhrmann et al., 1996), it may also modulate fluid retention that naturally occurs during the menstrual cycle (Foidart, 2005; Fenton et al., 2007; Bitzer and Paoletti, 2009). Drospirenone possesses spironolactone-derived anti-androgenic and anti-mineralocorticoid receptor properties without concomitant glucocorticoid receptor activity (Schindler et al., 2003). As such, drospirenone-containing contraceptives are FDA-approved to treat acne vulgaris and PMDD, a mood disorder different from premenstrual syndrome affecting approximately 5% of women (Fenton et al., 2007; Hofmeister and Bodden, 2016). Although drospirenone was reported to increase deep vein thrombosis when it was first popularized, this finding has since been refuted, and its safety profile is considered consistent with other clinically-used progestins (Larivée et al., 2016). It remains a popular progestin component in both oral contraceptives (e.g., YAZ^®^, Yazmin^®^, Ocella™, Slynd^®^, and Nextsellis^®^) and menopausal hormone therapies (e.g., Angeliq^®^) at the time of this writing.

Though drospirenone and EE have been reported to improve anxiety, PMDD symptoms, and psychosexual wellbeing in reproductive age women (Paoletti et al., 2004; Pearlstein et al., 2005; Yonkers et al., 2005; Nappi et al., 2009) and drospirenone plus E2 was shown to not impact cognition in menopausal women (Davison et al., 2013), little attention has been dedicated to methodically understanding drospirenone’s impact on memory and anxiety-like behaviors from a preclinical perspective. Given drospirenone’s unique pharmacological properties and potential for alleviating cognitive symptoms associated with PMDD, it is of significant interest to evaluate its effects on cognition alone and in combination with the synthetic estrogen EE. Our laboratory has demonstrated unique cognitive effects of progestins and estrogens depending on whether the drugs are administered alone or in combination with each other, and which animal model is used to evaluate these effects. For example, the progestin levonorgestrel has null or beneficial effects on spatial working memory when given alone (Braden et al., 2017; Prakapenka et al., 2018), but its beneficial effect is attenuated when combined with E2 in middle-aged ovariectomized (Ovx; surgical ovary removal) rats (Prakapenka et al., 2018). Yet, when E2 and levonorgestrel were co-administered to middle-aged rats with intact but follicle-depleted ovaries, this combined treatment regimen benefited spatial memory, anxiety-like, and depressive-like behaviors (Koebele et al., 2021a). Our laboratory has also reported spatial memory impairments associated with the progestins segesterone acetate (Woner et al., 2019) and medroxyprogesterone acetate (Braden et al., 2010, 2011).

Estrogens, progesterone, and their synthetic analogs, impact multiple neural systems to influence learning and memory processes. For example, both E2 (Nakamura et al., 2004; Joh et al., 2006) and the progestin medroxyprogesterone acetate (Pazol et al., 2009; Braden et al., 2010) act on the λ-aminobutyric acid (GABA)ergic system, which is the primary inhibitory neurotransmitter system in the brain and a critical modulator of normal learning and memory. Indeed, E2 is known to modulate hippocampal GABAergic activity (Murphy et al., 1998; Nakamura et al., 2004; Moura and Petersen, 2010; Wójtowicz and Mozrzymas, 2010) and synaptic plasticity (Woolley and McEwen, 1993, 1994; Woolley et al., 1997; Miranda et al., 1999; Barha and Galea, 2010; Frankfurt and Luine, 2015; Smith et al., 2016). Although the hippocampus is the most well-characterized structure in terms of E2’s impact on cognition, newer studies implicate ovarian hormones in functional changes in the perirhinal and entorhinal cortices, including E2-induced decreases in perirhinal cortex dendritic spine density (Gervais et al., 2015). Recently, it was shown that membrane-bound estrogen receptors enhanced synaptic excitation in the entorhinal cortex, while progesterone and allopregnanolone did not alter entorhinal synaptic responses (Batallán Burrowes et al., 2021). Systemic and intracranial E2 infusions into the perirhinal and entorhinal cortices enhanced novelty preference in Ovx rats, but impaired delayed-non-match to sample task performance (Petrulis and Eichenbaum, 2003; Gervais et al., 2013, 2016), demonstrating hormone-mediated cognitive-behavioral implications. We have previously reported altered glutamate decarboxylase (GAD)65+GAD67 expression in the hippocampus and surrounding cortical areas of Ovx rats treated with medroxyprogesterone acetate (Braden et al., 2010), which points to a putative mechanism for the detrimental cognitive effects of progestogens. Natural progesterone can reverse estrogen-induced growth factor increases in the entorhinal cortex in aged female rats (Bimonte-Nelson et al., 2004a), while the progestin segesterone acetate increased expression of insulin-like growth factor-1 receptor (IGF1-R), which is important for neurogenesis, in the frontal cortex of mice (Chen et al., 2018). Estrogen receptors also frequently colocalize with IGF1-R receptors on neuronal and glial cells, which may promote estrogen-induced neuroprotective effects (Garcia-Segura et al., 2007). Indeed, even short-term E2 administration has been shown to produce long-lasting increases in IGF1-R expression and benefit cognition (Witty et al., 2013), while IGF1-R blockade alters E2-induced hippocampal plasticity changes and spatial memory enhancements (Nelson et al., 2014). While evaluations of neurobiological mechanisms of the synthetic analog EE are in their infancy, our laboratory reported dose-dependent effects of EE on memory, wherein a higher dose impaired memory (Mennenga et al., 2015a). This underscores the need to investigate neural actions of EE, as well as whether drospirenone has similar neurobiological actions as other progestogen-mediated effects reported in the literature.

Elucidating whether drospirenone differentially impacts cognition in contrast to progestins from traditional derivatives is an important step toward refining and discovering novel pharmacotherapies that can provide long-term health benefits, including potential neuroprotection. In order to assess the impact of drospirenone on cognition, two experiments were performed. Using young adult, Ovx Fischer-344-CDF (F344-CDF) rats, Experiment 1 evaluated a range of doses of drospirenone to determine an optimal dosing regimen that impacted cognition. Experiment 2 incorporated the optimal dose determined from Experiment 1 and combined this dose with EE to investigate cumulative effects of the drugs on cognitive performance. Brains from both experiments were analyzed for GAD65, GAD67, and IGF1-R protein expression.

## Materials and Methods

Experimental procedures and statistical analyses were identical for Experiment 1 and Experiment 2.

### Subjects

One hundred sexually inexperienced 3-month-old female F344-CDF rats were obtained from Charles-River Laboratories (Raleigh, NC, United States). Forty subjects were included in Experiment 1 and 60 subjects were included in Experiment 2. Upon arrival to the animal facility, all rats were pair-housed, provided with free access to food and water for the duration of the experiment, and were maintained on a 12-h light/dark cycle for the entirety of the experiment. Procedures were approved by the Arizona State University Institutional Animal Care and Use Committee and adhered to National Institutes of Health standards.

### Ovariectomy

After 9 ± 1 day of acclimation to the vivarium, all rats underwent Ovx in order to initiate a “blank ovarian hormone slate,” which permits the evaluation of specific treatment effects without interactions with endogenously circulating hormones. Rats were anesthetized via inhaled isoflurane anesthesia. Five mg/kg/mL of the NSAID carprofen (Rimadyl^®^; Pfizer Pharmaceutical, Hospira Inc., Lake Forest, IL, United States) was administered to prevent post-surgical discomfort. Following sterilization of the surgical area, all rats received bilateral dorsolateral incisions through the skin and muscle. Ovaries and tips of the uterine horns were ligated and excised on each side. Muscle was sutured with dissolvable Vicryl suture and a local anesthetic, bupivacaine (Marcaine^®^; Pfizer Pharmaceutical, Hospira Inc., Lake Forest, IL, United States), was applied topically to the incision site. Skin was sutured with dissolvable Vicryl suture. All rats received two mL of sterile saline subcutaneously to prevent post-surgical dehydration.

### Hormone Treatment: Experiment 1

Drospirenone is abbreviated as DRSP in reference to treatment groups. Rats were randomly assigned to one of the following treatment groups (*n* = 10/group): Vehicle (sesame oil, control), DRSP-Low (12.5 μg/day), DRSP-Medium (30 μg/day), or DRSP-High (300 μg/day). Two ± one days after Ovx surgery, daily subcutaneous treatment injections began. Each treatment was administered in 0.1 mL of sesame oil in the scruff of the neck, and continued throughout the entirety of the experiment until euthanasia. The low DRSP dose was based on the most common dose prescribed to women in a combined oral contraceptive (3 mg/day), adjusted for an average weight (250 *g*) Ovx rat. Clinically, the ratio of DRSP to EE in a typical combined oral contraceptive is 100:1, so the medium DRSP dose reflected this ratio when considering the range of EE doses used in Experiment 2. The high DRSP dose was a replication of the dose used in a prior study from another laboratory (Muhn et al., 1995) that resulted in ovulation inhibition in ovary-intact adult rats.

### Hormone Treatment: Experiment 2

Rats were randomly assigned to one of the following treatment groups (*n* = 10/group): Vehicle (sesame oil, control); DRSP (30 μg DRSP/day); EE-Low (0.125 μg EE/day), EE-High (0.3 μg EE/day), DRSP + EE-Low (30 μg DRSP/day + 0.125 μg EE), or DRSP + EE-High (30 μg DRSP/day + 0.3 μg EE). All treatments were administered in the same fashion as Experiment 1, in 0.1 mL of sesame oil in the scruff of the neck beginning 2 ± 1 days after Ovx and continued daily throughout the experiment until euthanasia. The EE doses utilized were based on prior research from our laboratory; the Low-EE dose represented a typical dose of EE in a modern-day oral contraceptive (30–35 μg/day), and the High-EE dose represented the higher doses of EE prescribed in earlier generations of oral contraceptives (75–80 μg/day), each adjusted for rat body weight (Mennenga et al., 2015a). The DRSP-Medium dose (30 μg/day) was chosen for use in Experiment 2 based on Experiment 1 results.

### Vaginal Cytology

Eighteen days after the first hormone injection, vaginal smears were performed for three consecutive days to confirm successful Ovx in Vehicle-treated rats and evaluate how DRSP and/or EE treatment impacted vaginal epithelial cells after Ovx. Cytology was characterized based on specifications in Goldman et al. (2007) and Koebele and Bimonte-Nelson (2016), whereby: diestrus smears contained leukocytes with or without the presence of cornified cells; proestrus smears contained round, nucleated epithelial cells and cornified cells present in clusters; estrus was defined by the presence of cornified cells; and metestrus contained a combination of cornified cells, leukocytes, round cells, and keratinized, needle-like cells (Goldman et al., 2007; Koebele and Bimonte-Nelson, 2016).

### Body Weights

Beginning at Ovx surgery (baseline), weekly weights (grams) were recorded for all rats until the end of each experiment.

### Behavioral Battery

One month after daily hormone treatment initiation, rats were assessed on the water radial-arm maze (WRAM) and Morris water maze (MM) to evaluate spatial working and reference memory (RM). Following water maze tasks, rats were tested on the open field task (OFT) to assess locomotor activity and anxiety-like behavior.

### Water Radial-Arm Maze

The WRAM was an eight arm apparatus used to test spatial working and RM in rodents, as previously described (Bimonte and Denenberg, 1999; Bimonte et al., 2000; Bimonte-Nelson et al., 2015). Working memory required updating within a session. RM remained constant through the entirety of the task across days. Each arm was identical in size (29.7 cm long × 12.7 cm wide) and evenly spaced, radiating out from the circular center of the maze. Black non-toxic powdered paint was used to make the water (18–20°C) opaque. Four out of the eight arms contained hidden platforms placed 2 cm below the water’s surface at the beginning of each daily testing session. The specific locations of the platforms were constant within a rat for all testing days, but platform location combinations varied among rats and combinations were counterbalanced across treatment groups. Salient spatial cues were present on the walls around the maze to assist with spatial navigation.

Rats underwent baseline WRAM testing for 12 consecutive days, with four trials administered per daily testing session (one trial per hidden platform). The trial began when the experimenter placed the rat in the non-platformed start arm. Rats had 3 min per trial to locate a hidden platform. If the rat did not locate a platform in the maximum allotted time of 3 min, the experimenter led the rat to the nearest hidden platform. Once a platform was located, the rat was permitted to stay on it for 15 s, and then the experimenter removed the rat from the maze and placed it back into a heated testing cage for a 30 s inter-trial-interval. During those 30 s, the experimenter removed the just-found platform from the maze for the remainder of the daily testing session and gently stirred the water with a net to obscure potential olfactory cues and remove any debris from the water. The rat was placed back into the maze for the remaining three trials in an identical manner. Following 12 days of baseline testing, on day 13, a 6-h delay was implemented between trials two and three to test delayed working memory retention. Cognitive performance on the WRAM was quantified by the number of non-platformed arm entries—called errors—committed prior to locating a platform on each trial. An arm entry was quantified when the rat’s nose passed a designation mark 11 cm into the arm that was visible to the experimenter but not visible to the rat. Errors were defined in one of three categories: working memory correct (WMC) errors were entries into a previously platformed arm (which may occur on trials 2–4), RM errors were entries into a never-platformed arm for the first time within a daily testing session (capped at four errors), and working memory incorrect (WMI) errors were defined as repeat entries into never-platformed arms within a daily testing session.

### Morris Water Maze

The MM was a large round tub (diameter = 188 cm) filled with 18–20°C black-painted water used to assess spatial RM (Morris et al., 1982; Bimonte-Nelson et al., 2015; Morris, 2015). One hidden platform was submerged within the northeast quadrant of the maze, where it remained for all days and trials. Spatial cues were placed on the walls in the testing room to aid in spatial navigation. Each rat received four trials per day for five days. On each trial, a rat was dropped off from a cardinal direction (north, east, south, or west). The order of drop-off locations was the same for all rats within a day, but changed across days. The maximum trial time was 60 s. If a rat did not locate the hidden platform within the maximum allotted time, the experimenter led the rat to the platform. Once the rat found the platform, it remained on the platform for 15 s prior to being returned to its heated testing cage for an inter-trial-interval of approximately 15 min. The rats’ swim paths were recorded using Ethovision software (Noldus Instruments, Wageningen, Netherlands). On the fifth testing day, an additional trial was implemented following the four baseline trials. During this trial, called the probe trial, the platform was removed from the maze and the rats were allowed to swim freely for 60 s to assess spatial localization to the platform.

### Open Field Task

The OFT measured locomotor activity and anxiety-like behavior. This task has been shown to be sensitive to the presence and absence of ovarian hormones (Hiroi and Neumaier, 2006). The OFT was a 100 cm × 100 cm × 30 cm black plexiglass arena. Although some paradigms use a bright light in the center of the maze, this assay was completed in red light (i.e., darkness for rats), as we have previously published (Koebele et al., 2021a). This is because rats with significant anxiety-like phenotypes tend not to move at all if the center of the arena is lit. One day prior to the OFT, the arena was cleaned with an enzyme cleaner to remove any odors in and on the box. Rats were acclimated to the anteroom of the testing area for at least 30 min. Each rat was placed in the arena along the north wall. The experimenter quietly exited the room while the rat was allowed to explore the arena freely for a 10-min trial. The rat was then placed back in its testing cage and removed from the room. The experimenter counted and removed any fecal boli from the arena, cleaned the arena with water, and dried it with paper towel prior to the next subject’s trial. Dependent variables assessed in the OFT were total distance moved, as well as distance moved and time spent in the arena center (inner nine squares), small center (inner-most square), and arena corners. Twenty-five evenly spaced squares were digitally overlaid on the OFT tracks, and distance moved and time spent were recorded using Ethovision tracking software.

### Euthanasia

One day following OFT completion, rats were deeply anesthetized with inhaled isoflurane anesthesia. Brains were removed and the dorsal hippocampus, entorhinal cortex, and perirhinal cortex of the right hemisphere were rapidly raw dissected, weighed, and frozen at −70°C until western blot analysis. Ovx status was verified at necropsy and the uterine horns were removed from the body cavity, trimmed of visible fat, and wet weight was recorded.

### Western Blot Protein Analysis

Right hemisphere dorsal hippocampus, entorhinal cortex, and perirhinal cortex from each experiment were analyzed for GAD65 expression, GAD67 expression, and IGF1-R expression via western blots. Frozen raw tissue samples were suspended in a 1:25 weight-to-volume RIPA buffer solution [150 mM NaCl, 1%Triton X-100, 0.1% SDS, 0.5% sodium deoxycholate, 50-mM Tris–HCl, protease inhibitor (Millipore-Sigma, CAT#5892791001)], and phosphatase inhibitor (Millipore-Sigma, CAT#524625). Tissues were kept on ice at all times and homogenized using a probe sonicator (Ultrasonic Processor, Cole Parmer, IL, United States), and then centrifuged at 10,000 rpm for 10 min at 4°C. Cleared supernatants were collected, aliquoted, and frozen at −70°C until analysis. Bicinchoninic acid protein assays (Thermo-Fisher Scientific, Pittsburgh, PA, United States) were used to determine sample protein concentrations.

Within an experiment, treatment groups were counterbalanced and equally represented on each gel run. The NuPAGE PowerEase electrophoresis system was utilized for tissue processing. Tissue samples for each region were loaded at an equal protein concentration and were run on a 4–12% NuPAGE Bis-Tris gel in an XCell SureLock Mini-Cell with MOPS running buffer (Invitrogen, Carlsbad, CA, United States) and transferred to an Immobilon polyvinylidene difluoride membrane. The membrane was washed in 1× Tris-buffered saline with 0.1% Tween (TBST) and blocked in 5% non-fat milk for 1 h at room temperature. Following blocking, the membrane was washed in 1× TBST and incubated overnight on a shaker at 4°C with anti-GAD65 (1:5000; Abcam, ab26113; 65 kDa), anti-GAD67 (1:10,000; Abcam, ab 26116; 67 kDa), anti-IGF1-R (1:1000; Cell Signaling, #9750S; 95 kDa), and loading control anti-beta-actin (1:20,000; Cell Signaling, #4970S; 45 kDa) in 5% non-fat milk. The following day, the membrane was washed in 1× TBST and incubated with secondary antibodies anti-mouse horseradish peroxidase (HRP; 1:2000; Cell Signaling #7076S) for GAD65 and GAD67, and anti-rabbit HRP (1:2000; Cell Signaling #7074) for IGF1-R and beta-actin for 1 h at room temperature in 5% non-fat milk. The membrane was washed in 1× TBST, and developed using chemiluminescence (Lumiglo and peroxide, Cell Signaling #7003S) in a film developer (Konica SRX-101A Film Processor, Tokyo, Japan). Films were scanned to the computer as JPEG files at 600 dpi. Densitometry analyses were completed using ImageJ software (Gallo-Oller et al., 2018). GAD65, GAD67, and IGF1-R bands were normalized to corresponding beta-actin for each blot.

### Statistical Analyses

*A priori* two-group comparisons between each DRSP group and Vehicle were completed using repeated measures analysis of variance (ANOVA) for Experiment 1. For Experiment 2, each DRSP, EE, and DRSP + EE group was compared to Vehicle using two-group comparisons. Additionally, we compared EE-Low and EE-High groups to each other to assess dose-dependent effects, as well as compared EE-Low, EE-High, and DRSP groups to the combinations of DRSP + EE to assess effects of the hormones alone vs in combination with one another to evaluate differential cognitive effects when hormone treatments are co-administered (Prakapenka et al., 2018; Koebele et al., 2021a). Alpha level was set to 0.05 for all analyses. Generalized eta squared (η_*G*_^2^) was calculated for repeated measures ANOVA as a measure of effect size (Olejnik and Algina, 2003; Bakeman, 2005). For all non-repeated measures ANOVA, eta squared (η^2^) was reported. Standard effect size guidelines were applied to interpretations of η_*G*_^2^ and η^2^, with 0.02 as a small effect, 0.13 as a medium effect, and 0.26 as a large effect (Olejnik and Algina, 2003; Bakeman, 2005).

Water radial-arm maze data were separated into three phases based on error-making patterns in the learning curve, as we have previously published (Mennenga et al., 2015b; Braden et al., 2017; Prakapenka et al., 2018; Koebele et al., 2019, 2021a). Day 1 was considered training and was excluded from the analysis. Days 2–5 were the Early Acquisition Phase when rats are exploring the maze and learning the rules of the task. Days 6–9 were the Late Acquisition Phase, when error scores begin to decrease but there is still variability in performance as rats consolidate the win-shift rules, such that they have to shift spatial locations to be rewarded (i.e., removed from the maze) on each trial. Days 10–12 were the Asymptotic Phase, when rats are reaching peak performance and approaching asymptotic error scores. WMC, RM, and WMI errors were the dependent measure analyzed separately for the Early Acquisition Phase, Late Acquisition Phase, and Asymptotic Phase. Trials (three trials for WMC, four trials for RM and WMI) were nested within days as repeated measures. Treatment was the independent variable. Based on prior findings indicating working memory load-dependent hormone effects, we also analyzed the moderate (Trial 3) and maximum (Trial 4) working memory load trials separately, as previously done (Braden et al., 2010; Mennenga et al., 2015a; Koebele et al., 2017, 2019, 2021b; Prakapenka et al., 2018).

Morris water maze data were analyzed using repeated measures ANOVA, with Treatment as the independent variable and Swim Distance to Platform (cm) as the dependent variable, with four trials nested within the five days as repeated measures for each two-group comparison. Groups were evaluated separately for probe trial performance [percent of total swim distance in the northeast (previously platformed) Quadrant vs the southwest (opposite) Quadrant].

Open field task data were analyzed using ANOVA, with Treatment as the independent variable, and Total Distance Moved (cm), Center Distance, Center Time, Small Center Distance, Small Center Time, Corner Distance, and Corner Time as dependent variables for each two-group comparison.

Western blot protein analyses were completed using ANOVA, with Treatment as the independent variable and GAD65, GAD67, and IGF1-R expression normalized to beta-actin (arbitrary units; AU) in the dorsal hippocampus, entorhinal cortex, and perirhinal cortex as the dependent variable for each two-group comparison. Body weight and uterine weight were also analyzed using ANOVA, with Treatment as the independent variable and Weight (*g*) as a dependent variable for each two-group comparison.

Pearson’s r correlations were completed between IGF1-R expression, GAD65 expression, and GAD67 expression in the dorsal hippocampus, entorhinal cortex, and perirhinal cortex with WMC and WMI errors on the WRAM for each phase across all trials, as well as for Trial 3 only and Trial 4 only. The False Discovery Rate (FDR) correction using the Benjamini-Hochberg procedure was applied with an FDR = 0.25 (Benjamini and Hochberg, 1995; McDonald, 2014).

## Results

### Experiment 1

#### Water Radial-Arm Maze

##### Early Acquisition Phase

**FIGURE 1:**
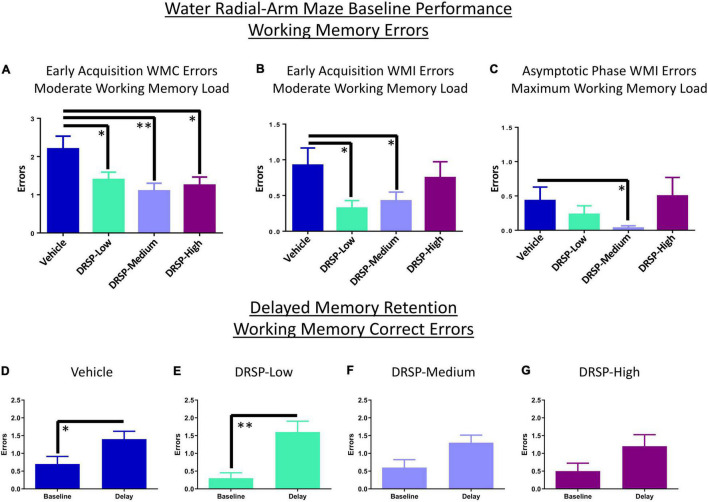
Experiment 1 Water radial-arm maze. **(A)** During Early Acquisition, all groups treated with drospirenone had improved WMC performance compared to rats without hormone treatment when working memory load was moderately taxed. **(B)** This working memory benefit was extended to WMI errors on the moderate working memory load trial for DRSP-Low and DRSP-Medium groups compared to rats without hormone treatment. **(C)** During the Asymptotic Phase, DRSP-Medium continued to enhance WMI performance compared to rats without hormone treatment on Trial 4, the maximum working memory load trial. Rats treated with Vehicle **(D)** or DRSP-Low **(E)** showed impaired delayed memory retention on Trial 3 following a 6-h delay compared to the previous day’s baseline performance. Rats treated with **(F)** DRSP-Medium and **(G)** DRSP-High treatment did not exhibit a statistically significant delay-related impairment. *p < 0.05 and ^**^p < 0.01. Vehicle *n* = 10, DRSP-Low *n* = 10, DRSP-Medium *n* = 10, and DRSP-High *n* = 10.

Across all days and trials of the Early Acquisition Phase, there was a main effect of Treatment for the Vehicle vs DRSP-Low comparison [*F*_(1_,_18)_ = 5.71, *p* < 0.05, η_*G*_^2^ = 0.02] where rats treated with DRSP-Low made fewer WMC errors on Trials 2–4 compared to Ovx rats without hormone treatment. All DRSP-treated groups made fewer errors than Vehicle-treated rats on the moderate working memory load trial (Vehicle vs DRSP-Low: [*F*_(1_,_18)_ = 5.69, *p* < 0.05, η_*G*_^2^ = 0.06]; Vehicle vs DRSP-Medium: [*F*_(1_,_18)_ = 10.50, *p* < 0.01, η_*G*_^2^ = 0.10]; and Vehicle vs DRSP-High: [*F*_(1_,_18)_ = 7.57, *p* < 0.05, η_*G*_^2^ = 0.07]; Figure 1A). A Treatment main effect was also present for WMI errors on the moderate memory load trial for the Vehicle group vs the DRSP-Low group [*F*_(1_,_18)_ = 7.83, *p* < 0.05, η_*G*_^2^ = 0.08] and vs the DRSP-Medium group [*F*_(1_,_18)_ = 5.13, *p* < 0.05, η_*G*_^2^ = 0.05], where each DRSP-treated group made fewer WMI errors compared to Vehicle-treated rats (Figure 1B). There were no differences in working memory performance on the maximum working memory load trial during early acquisition, nor were there RM effects in the Early Acquisition Phase.

##### Late Acquisition Phase

There were no main effects of Treatment during the Late Acquisition Phase for WMC, WMI, or RM errors for any two-group comparison.

##### Asymptotic Phase

There was a main effect of Treatment for WMI errors for the Vehicle vs DRSP-Medium comparison [*F*_(1_,_18)_ = 4.47, *p* < 0.05, η_*G*_^2^ = 0.02] and a Trial × Treatment interaction for this comparison [*F*_(3_,_54)_ = 4.47, *p* < 0.01, η_*G*_^2^ = 0.06] across all trials and days of the Asymptotic Phase. On the maximum working memory load trial, DRSP-Medium rats made fewer WMI errors compared to Vehicle treated rats ([*F*_(1_,_18)_ = 4.47, *p* < 0.05, η_*G*_^2^ = 0.07], Figure 1C). There were no significant effects for WMC or RM errors in the Asymptotic Phase.

##### Delayed Memory Retention

Working memory correct errors on Trial 3 from the final day of baseline testing (day 12) were compared to WMC errors on Trial 3 after the 6-h delay (the first post-delay trial on day 13) for each treatment group. Post-delay errors on Trial 3 were increased for Vehicle rats [*F*_(9_,_1)_ = 5.44, *p* < 0.05, η_*G*_^2^ = 0.52] and DRSP-Low rats [*F*_(9_,_1)_ = 10.79, *p* < 0.01, η_*G*_^2^ = 0.33] compared to the previous day’s performance in each group (Figures 1D,E). DRSP-Medium and DRSP-High groups did not show a delay-induced impairment (Figures 1F,G).

#### Morris Water Maze

**FIGURE 2:**
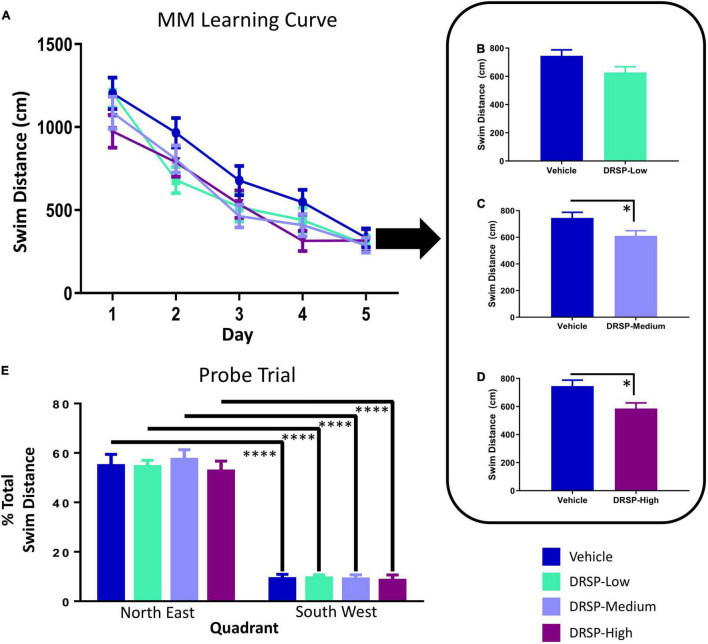
Experiment 1 Morris water maze. Across all days of testing **(A)**, rats treated with the DRSP-Medium dose **(C)** and the DRSP-High dose **(D)** swam less distance to reach the platform compared to rats without hormone treatment, while the DRSP-Low group **(B)** performed similarly to the Vehicle group. **(E)** On the probe trial, all groups localized to the previously platformed target quadrant. *p < 0.05 and ^****^p < 0.0001. Vehicle *n* = 10, DRSP-Low *n* = 10, DRSP-Medium *n* = 10, and DRSP-High *n* = 10.

Across all days and trials, there was a main effect of Treatment for the Vehicle vs DRSP-Medium comparison [*F*_(1_,_18)_ = 7.37, *p* < 0.05, η_*G*_^2^ = 0.02], and for the Vehicle vs DRSP-High comparison [*F*_(1_,_18)_ = 7.21, *p* < 0.05, η_*G*_^2^ = 0.03] where rats treated with the medium or high dose of drospirenone swam less distance to the platform compared to Vehicle-treated rats (Figures 2A–D). Each group was analyzed separately on the probe trial. There was a Quadrant main effect for each group (Vehicle: [*F*_(9_,_1)_ = 60.17, *p* < 0.0001, η_*G*_^2^ = 0.23]; DRSP-Low: [*F*_(9_,_1)_ = 174.65, *p* < 0.0001, η_*G*_^2^ = 0.27]; DRSP-Medium: [*F*_(9_,_1)_ = 78.85, *p* < 0.0001, η_*G*_^2^ = 0.18]; and DRSP-High: [*F*_(9_,_1)_ = 56.32, *p* < 0.0001, η_*G*_^2^ = 0.15]). This indicated that all rats, regardless of treatment, swam a greater percent of total distance in the target, compared to the opposite, quadrant (Figure 2E).

#### Open Field Task

DRSP administration at any dose had no impact on overall locomotor activity or measures of anxiety-like behavior in Ovx rats as measured in the OFT.

#### Peripheral Markers of Hormone Stimulation

##### Vaginal Smears

All groups displayed diestrus-like or blank cytology, indicating that Ovx was successful and that daily drospirenone treatment at all doses evaluated did not stimulate the vaginal epithelium following Ovx.

##### Body Weight and Uterine Weights

There were no main effects of Treatment on body or uterine weights at euthanasia for any two-group comparison, indicating that drospirenone administration at any assessed dose did not influence body or uterine weight after Ovx.

#### Western Blot Protein Analysis

**FIGURE 3:**
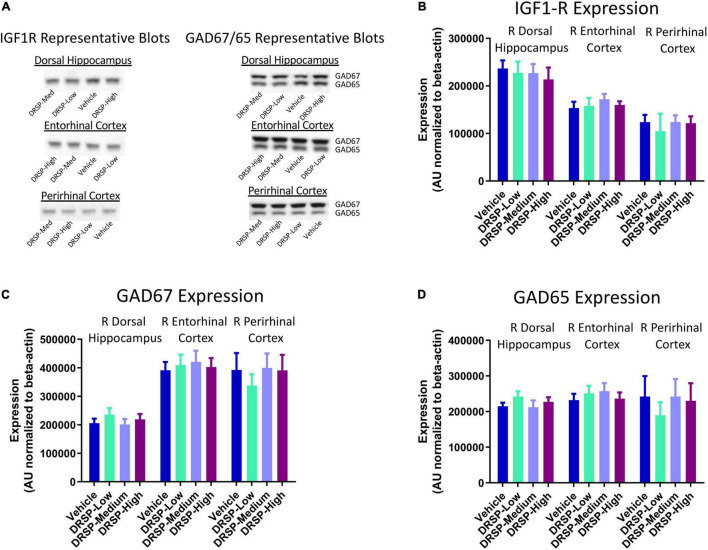
Experiment 1 Western blot protein analysis. **(A)** Representative images of IGF1-R, GAD65, and GAD67 western blot analysis within the dorsal hippocampus, entorhinal cortex, and perirhinal cortex. There were no Treatment differences in IGF1-R expression **(B)**, GAD67 expression **(C)**, or GAD65 expression **(D)** for any brain region evaluated. Vehicle *n* = 10, DRSP-Low *n* = 10, DRSP-Medium *n* = 10, and DRSP-High *n* = 10.

Representative blots for each protein within all assessed brain regions are pictured in Figure 3A. There were no effects of Treatment for GAD65 expression, GAD67 expression, or IGF1-R expression in the dorsal hippocampus, entorhinal cortex, or perirhinal cortex for any two-group comparison (Figures 3B–D), indicating the drospirenone treatment after Ovx did not impact GAD or IGF1-R protein expression in brain regions important for spatial learning and memory.

#### Correlations

After correcting for multiple comparisons using the FDR method, there were no significant correlations between western blot results and WRAM performance in Experiment 1.

### Experiment 2

#### Water Radial-Arm Maze

##### Early Acquisition Phase

**FIGURE 4:**
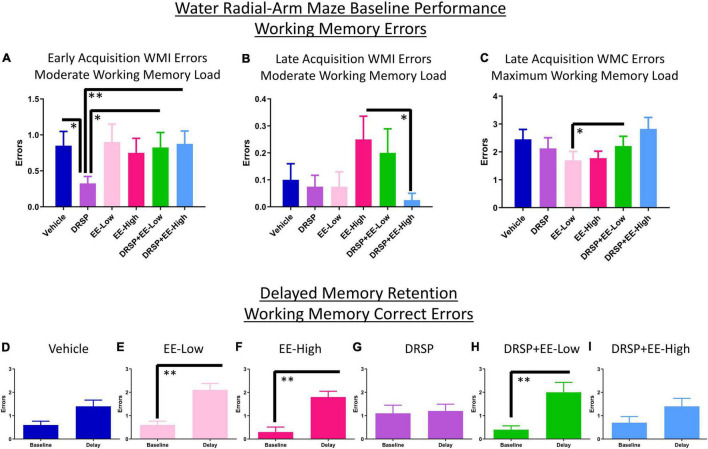
Experiment 2 Water radial-arm maze. **(A)** During early acquisition, rats treated with drospirenone only showed enhanced WMI performance on the moderate working memory load trial compared to rats without hormone treatment, or rats given a combination of drospirenone plus a low or high dose of EE. **(B)** During the Late Acquisition Phase, rats treated with a combination of drospirenone and high EE had enhanced WMI performance on the moderate working memory load trial compared to rats treated with a high dose of EE only. **(C)** Rats treated with a combination of drospirenone and low EE showed impaired performance on the maximum working memory load trial compared to a low dose of EE alone during Late Acquisition. **(D)** Rats without hormone treatment did not exhibit a delay-induced memory impairment, **(E,F,H)** while rats treated with EE-Low, EE-High, and DRSP + EE-Low were significantly impaired following a 6-h delay. **(G,I)** The DRSP group and the DRSP + EE-High group were not significantly impaired following a 6-h delay. *p < 0.05 and ^**^p < 0.01. Vehicle *n* = 10, DRSP *n* = 10, EE-Low *n* = 10, EE-High *n* = 10, DRSP + EE-Low *n* = 10, and DRSP + EE-High *n* = 10.

There was a Trial × Treatment interaction for WMI errors for the Vehicle vs EE-Low group [*F*_(3_,_54)_ = 3.03, *p* < 0.05, η_*G*_^2^ = 0.01]. Rats treated with DRSP + EE-Low made fewer RM errors during early acquisition compared to Vehicle-treated rats [*F*_(1_,_18)_ = 5.17, *p* < 0.05, η_*G*_^2^ = 0.01]. DRSP-treated rats made fewer WMI errors than Vehicle-treated counterparts on the moderate working memory load trial alone [*F*_(1_,_18)_ = 7.03, *p* < 0.05, η_*G*_^2^ = 0.08], replicating findings from Experiment 1 (Figure 4A). Furthermore, rats treated with DRSP alone made fewer WMI errors on the moderate working memory load trial compared to DRSP + EE-Low rats [*F*_(1_,_18)_ = 7.79, *p* < 0.05, η_*G*_^2^ = 0.07] and DRSP + EE-High rats [*F*_(1_,_18)_ = 10.23, *p* < 0.01, η_*G*_^2^ = 0.09], indicating that the addition of EE at either dose impaired working memory compared to drospirenone alone during the Early Acquisition Phase (Figure 4A).

##### Late Acquisition Phase

Across all days and trials in the Late Acquisition Phase, the EE-Low vs DRSP + EE-Low comparison revealed a main effect of Treatment [*F*_(1_,_18)_ = 5.09, *p* < 0.05, η_*G*_^2^ = 0.04] as well as a Trial × Treatment interaction [*F*_(2_,_36)_ = 3.79, *p* < 0.05, η_*G*_^2^ = 0.03] for WMC errors. A Trial × Treatment interaction for WMI errors was present for the Vehicle vs EE-High comparison [*F*_(3_,_54)_ = 4.54, *p* < 0.01, η_*G*_^2^ = 0.05] and for the EE-High vs DRSP + EE-High comparison [*F*_(3_,_54)_ = 4.28, *p* < 0.01, η_*G*_^2^ = 0.07]. The DRSP + EE-High group made fewer WMI errors on the moderate working memory load trial compared to EE-High alone [*F*_(1_,_18)_ = 5.65, *p* < 0.05, η_*G*_^2^ = 0.09], suggesting that after initial learning takes place, the addition of drospirenone to a high dose of EE may prevent working memory impairments compared to a high dose of EE alone when working memory is moderately taxed (Figure 4B). On the maximum working memory load trial, the DRSP + EE-Low-treated group made more WMC errors compared to the EE-Low alone group [*F*_(1_,_18)_ = 5.17, *p* < 0.05, η_*G*_^2^ = 0.07; Figure 4C].

##### Asymptotic Phase

Across all days and trials on the Asymptotic Phase, there was a Trial × Treatment interaction for RM errors for the EE-Low vs EE-High comparison [*F*_(3_,_54)_ = 3.17, *p* < 0.05, η_*G*_^2^ = 0.05]. When the moderate and maximum working memory load trials were evaluated separately, no statistically significant effects were revealed for working memory during the Asymptotic Phase for any planned comparison.

##### Delayed Memory Retention

Working memory correct errors on Trial 3 from the last day of regular WRAM testing (day 12) were compared to Trial 3 after the 6-h delay (first post-delay trial) for each treatment group. Post-delay errors on Trial 3 were increased for the EE-Low group [*F*_(9_,_1)_ = 19.29, *p* < 0.01, η_*G*_^2^ = 0.44; Figure 4E], the EE-High group [*F*_(9_,_1)_ = 16.20, *p* < 0.01, η_*G*_^2^ = 0.36; Figure 4F], and the DRSP + EE-Low group [*F*_(9_,_1)_ = 12.52, *p* < 0.01, η_*G*_^2^ = 0.50; Figure 4H] compared to the previous day performance of each respective group. Rats treated with Vehicle, DRSP, or DRSP + EE-High did not exhibit a significant delay-induced impairment (Figures 4D,G,I).

#### Morris Water Maze

**FIGURE 5:**
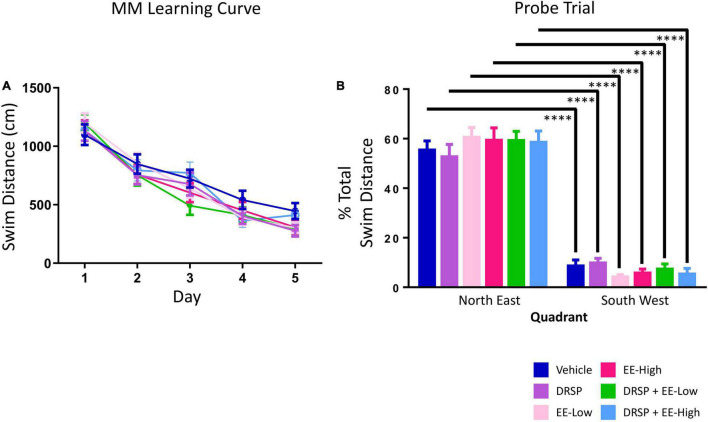
Experiment 2 Morris water maze. **(A)** There were no Treatment differences across MM Days 1–5. **(B)** All groups localized to the target quadrant during the probe trial. ^****^p < 0.0001. Vehicle *n* = 10, DRSP *n* = 10, EE-Low *n* = 10, EE-High *n* = 10, DRSP + EE-Low *n* = 10, and DRSP + EE-High *n* = 10.

There were no Treatment effects for any two-group comparisons, nor any Day × Treatment interactions (Figure 5A). There was a main effect of Quadrant for each group (Vehicle: [*F*_(9_,_1)_ = 63.43, *p* < 0.0001, η_*G*_^2^ = 0.13]; DRSP: [*F*_(9_,_1)_ = 40.74, *p* < 0.0001, η_*G*_^2^ = 0.21]; EE-Low: [*F*_(9_,_1)_ = 191.53, *p* < 0.0001, η_*G*_^2^ = 0.50]; EE-High: [*F*_(9_,_1)_ = 76.74, *p* < 0.0001, η_*G*_^2^ = 0.32]; DRSP + EE-Low: [*F*_(9_,_1)_ = 92.29, *p* < 0.0001, η_*G*_^2^ = 0.17]; and DRSP + EE-High: [*F*_(9_,_1)_ = 63.36, *p* < 0.0001, η_*G*_^2^ = 0.14]), with each treatment group swimming a greater percent of total distance in the previously platformed quadrant compared to the opposite quadrant (Figure 5B).

#### Open Field Task

**FIGURE 6:**
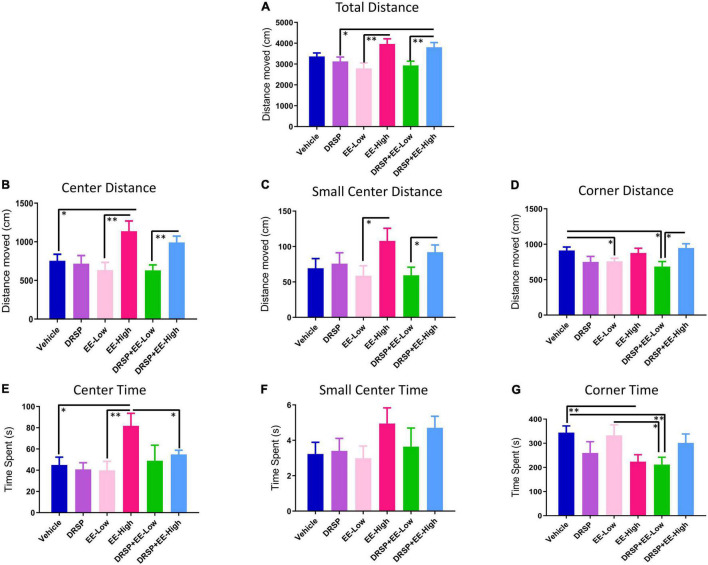
Experiment 2 Open field task performance. Performance varied across: **(A)** Total Distance Moved, **(B)** Center Distance, **(C)** Small Center Distance, **(D)** Corner Distance, **(E)** Center Time, **(F)** Small Center Time, and **(G)** Corner Time, in the OFT. In general, the EE-High group exhibited decreased anxiety-like behavior in the Open Field. *p < 0.05 and ^**^p < 0.01. Vehicle *n* = 10, DRSP *n* = 10, EE-Low *n* = 10, EE-High *n* = 10, DRSP + EE-Low *n* = 10, and DRSP + EE-High *n* = 10.

Total Distance Moved (cm) in the arena was a marker of locomotor activity (Figure 6A); a main effect of Treatment was observed for the EE-Low vs EE-High comparison [*F*_(1_,_18)_ = 10.77, *p* < 0.01, η^2^ = 0.60], with EE-High rats having a greater distance covered in the 10 min trial. Additionally, DRSP + EE-High rats covered a greater distance compared to the DRSP only group [*F*_(1_,_18)_ = 4.96, *p* < 0.05, η^2^ = 0.28] and compared to the DRSP + EE-Low group [*F*_(1_,_18)_ = 8.41, *p* < 0.01, η^2^ = 0.47]. EE-High rats covered more distance in the center of the arena, an indicator of anxiolytic behavior, compared to Ovx rats without hormone treatment [*F*_(1_,_18)_ = 5.92, *p* < 0.05, η^2^ = 0.33] and Ovx rats treated with a lower dose of EE [*F*_(1_,_18)_ = 9.14, *p* < 0.01, η^2^ = 0.51] (Figure 6B). The DRSP + EE-High group moved more than the DRSP + EE-Low group in the center of the arena [*F*_(1_,_18)_ = 11.28, *p* < 0.01, η^2^ = 0.63]. Small Center Distance (cm) was the distance traveled in the immediate center of the arena, and is an additional marker of anxiolytic behavior (Figure 6C). EE-High treated rats traveled more distance in the Small Center compared to EE-Low rats [*F*_(1_,_18)_ = 4.71, *p* < 0.05, η^2^ = 0.26]. DRSP + EE-High-treated rats traveled more distance in the Small Center compared to DRSP + EE-Low rats [*F*_(1_,_18)_ = 4.56, *p* < 0.05, η^2^ = 0.25]. Corner Distance analyses, a measure of anxiety-like behavior (Figure 6D), revealed the Vehicle group traveled more distance in the corners compared to the EE-Low group [*F*_(1_,_18)_ = 5.41, *p* < 0.05, η^2^ = 0.30], as well as the DRSP + EE-Low group [*F*_(1_,_18)_ = 6.99, *p* < 0.05, η^2^ = 0.39]. The DRSP + EE-High group moved more distance in the Corners compared to the DRSP + EE-Low group [*F*_(1_,_18)_ = 8.04, *p* < 0.05, η^2^ = 0.45].

For Time analyses in the OFT (Figure 6E), the EE-High group spent more time in the Center compared to the Vehicle group [*F*_(1_,_18)_ = 7.02, *p* < 0.05, η^2^ = 0.39], the EE-Low group [*F*_(1_,_18)_ = 8.40, *p* < 0.01, η^2^ = 0.47], and the DRSP + EE-High group [*F*_(1_,_18)_ = 4.69, *p* < 0.05, η^2^ = 0.26], indicating that EE-High treatment decreased anxiety-like behavior. Small Center time did not differ for any comparison (Figure 6F). For Corner Time (Figure 6G), Ovx rats without hormone treatment spent more time in the corners compared to the EE-High group [*F*_(1_,_18)_ = 9.05, *p* < 0.01, η^2^ = 0.50] and compared to the DRSP + EE-Low group [*F*_(1_,_18)_ = 10.42, *p* < 0.01, η^2^ = 0.58]. EE-Low rats spent more time in the corners than rats receiving the combination of DRSP + EE-Low treatment [*F*_(1_,_18)_ = 5.21, *p* < 0.05, η^2^ = 0.29]. Overall, Vehicle treatment was associated with increased anxiety-like behaviors, and EE-High treatment was associated with decreased anxiety-like behaviors.

#### Peripheral Markers of Hormone Stimulation

##### Vaginal Smears

The Vehicle group and the DRSP group exhibited blank or diestrus-like smears for all three days evaluated, indicating successful Ovx and a lack of stimulation from daily drospirenone treatment alone, replicating results from Experiment 1. Rats treated with EE-Low, EE-High, DRSP + EE-Low, and DRSP + EE-High exhibited cornified cells across all days, indicating EE-induced stimulation of the vaginal epithelium that was not qualitatively altered by concomitant drospirenone administration.

##### Body Weights and Uterine Weights

**FIGURE 7:**
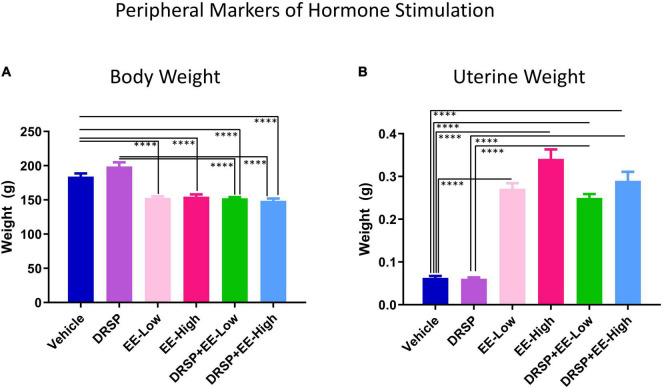
Experiment 2 Body weight and uterine weight. All subjects were ovariectomized. **(A)** Rats treated with EE alone and in combination with drospirenone weighed significantly less than rats without hormone treatment. Rats treated with drospirenone weighed more than those that received drospirenone plus EE at a low or high dose. **(B)** Uterine weights from rats treated with EE weighed significantly more at euthanasia compared to rats without hormone treatment. Rats treated with drospirenone had lower uterine weights compared to rats treated with a combination of drospirenone and EE, but did not differ from rats without hormone treatment. ^****^p < 0.0001. Vehicle *n* = 10, DRSP *n* = 10, EE-Low *n* = 10, EE-High *n* = 10, DRSP + EE-Low *n* = 10, and DRSP + EE-High *n* = 10.

Two-group comparisons were completed for body weight at the end of the experiment (Figure 7A). The Vehicle group weighed more than the EE-Low group [*F*_(1_,_18)_ = 36.26, *p* < 0.0001, η^2^ = 2.01], EE-High group [*F*_(1_,_18)_ = 26.24, *p* < 0.0001, η^2^ = 1.46], DRSP + EE-Low group [*F*_(1_,_18)_ = 40.02, *p* < 0.0001, η^2^ = 2.22], and DRSP + EE-High group [*F*_(1_,_18)_ = 36.61, *p* < 0.0001, η^2^ = 2.03]. The DRSP group weighed more than the DRSP + EE-Low group [*F*_(1_,_18)_ = 53.94, *p* < 0.0001, η^2^ = 3.00] and the DRSP + EE-High group [*F*_(1_,_18)_ = 51.22, *p* < 0.0001, η^2^ = 2.85]. Collectively, EE-treated groups, with and without concomitant drospirenone administration, did not differ from one another, suggesting that EE administration prevents weight gain in Ovx rats, and drospirenone treatment does not further alter body weight when combined with EE, at least with the current experimental parameters.

Wet uterine weight (*g*) was lower in the Vehicle group compared to the EE-Low group [*F*_(1_,_18)_ = 246.14, *p* < 0.0001, η^2^ = 13.56], EE-High group [*F*_(1_,_18)_ = 153.73, *p* < 0.0001, η^2^ = 8.62], DRSP + EE-Low group [*F*_(1_,_18)_ = 351.73, *p* < 0.0001, η^2^ = 19.4], and DRSP + EE-High group [*F*_(1_,_18)_ = 114.6, *p* < 0.0001, η^2^ = 6.45] (Figure 7B). Uterine weights in the Vehicle group and DRSP group did not differ from one another, replicating findings from Experiment 1. The DRSP group also had significantly lower uterine weights compared to the DRSP + EE-Low group [*F*_(1_,_18)_ = 386.84, *p* < 0.0001, η^2^ = 22.25] and DRSP + EE-High group [*F*_(1_,_18)_ = 118.16, *p* < 0.0001, η^2^ = 6.53]. Uteri from the EE-High group weighed more than the EE-Low group [*F*_(1_,_18)_ = 7.60, *p* < 0.01, η^2^ = 0.43]. Overall, the data indicate that unopposed EE-High treatment significantly increases uterine weights, while drospirenone treatment at the administered dose does not have a significant influence on uterine weights when given alone or combined with EE.

#### Western Blot Protein Analysis

**FIGURE 8:**
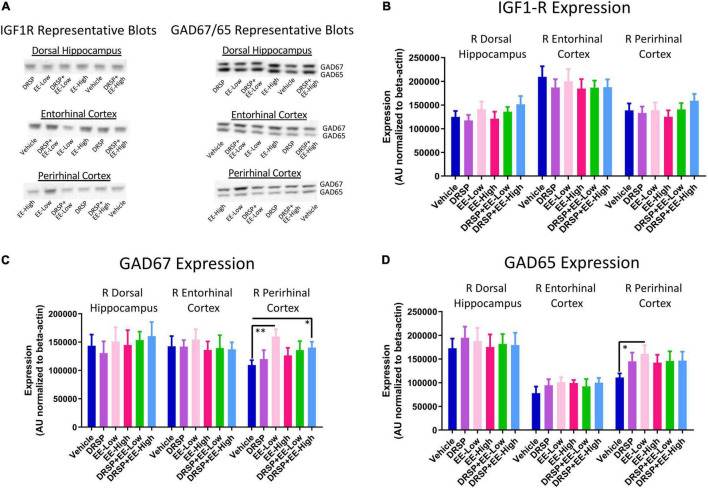
Experiment 2 Western blot protein analysis. **(A)** Representative images of IGF1-R, GAD65, and GAD67 western blot analysis within the dorsal hippocampus, entorhinal cortex, and perirhinal cortex. **(B)** There were no differences in IGF1-R expression between groups in any brain region evaluated. **(C)** Rats treated with a low dose of EE or drospirenone plus a high dose of EE showed increased GAD67 expression in the perirhinal cortex compared to rats without hormone treatment. No differences in GAD67 were detected between groups in the dorsal hippocampus or entorhinal cortex. **(D)** Rats treated with a low dose of EE had increased GAD65 expression in the perirhinal cortex compared to rats without hormone treatment. No differences in GAD65 were detected between groups in the dorsal hippocampus or entorhinal cortex. *p < 0.05 and ^**^p < 0.01. Vehicle *n* = 10, DRSP *n* = 10, EE-Low *n* = 10, EE-High *n* = 10, DRSP + EE-Low *n* = 10, and DRSP + EE-High *n* = 10.

Representative blots for each protein within all brain regions analyzed are pictured in Figure 8A. There were no differences in IGF1-R expression, GAD65 expression, or GAD67 expression for any comparison in the right dorsal hippocampus or right entorhinal cortex (Figures 8B–D). EE-Low treated-rats had increased GAD67 expression [*F*_(1_,_18)_ = 10.62, *p* < 0.01, η^2^ = 0.59; Figure 8C] and GAD65 expression [*F*_(1_,_18)_ = 5.96, *p* < 0.05, η^2^ = 0.33; Figure 8D] in the right perirhinal cortex compared to Vehicle-treated rats. DRSP + EE-High treated rats [*F*_(1_,_18)_ = 5.26, *p* < 0.05, η^2^ = 0.29] also exhibited increased GAD67 expression in the right perirhinal cortex compared to Vehicle-treated rats (Figure 8C). There were no differences in IGF-1R expression in the right perirhinal cortex (Figure 8B).

#### Correlations

**FIGURE 9:**
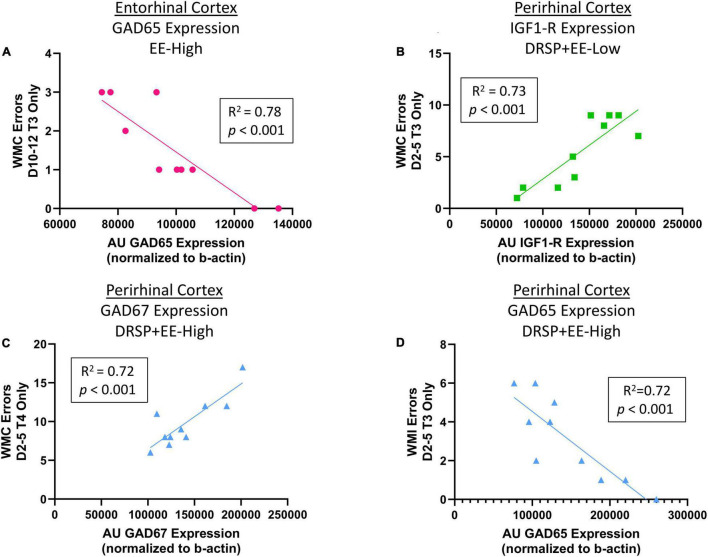
Experiment 2 Correlations between proteins of interest and WRAM performance. **(A)** Within the EE-High group, greater GAD65 expression in the entorhinal cortex was associated with fewer WMC errors when working memory was moderately taxed during the Early Acquisition Phase of WRAM. **(B)** Within the DRSP + EE-Low group, greater IGF1-R expression in the perirhinal cortex was associated with more WMC errors when working memory was moderately taxed during the Early Acquisition Phase of WRAM. **(C)** Within the DRSP + EE-High group, greater GAD67 expression in the perirhinal cortex was associated with increased WMC errors on the maximum working memory load trial during Early Acquisition. **(D)** Within the DRSP + EE-High group, greater GAD65 expression in the perirhinal cortex was associated with decreased WMI errors on the moderate working memory load trial during Early Acquisition. Vehicle *n* = 10, DRSP *n* = 10, EE-Low *n* = 10, EE-High *n* = 10, DRSP + EE-Low *n* = 10, and DRSP + EE-High *n* = 10.

After correction for multiple comparisons using the FDR method, results revealed a negative correlation for GAD65 expression in the right entorhinal cortex and WMC errors committed on Trial 3 during the Asymptotic Phase for the EE-High group, such that greater GAD65 expression was associated with fewer WMC errors on the moderate working memory load trial (*R*^2^ = 0.78, *p* < 0.001, Benjamini-Hochberg *p* value = 0.243, Figure 9A). Within the perirhinal cortex, the DRSP + EE-Low group showed a positive correlation between IGF1-R expression and WMC errors on Trial 3 during the Early Acquisition Phase, where greater IGF1-R expression was associated with more WMC errors (*R*^2^ = 0.73, *p* < 0.001, Benjamini-Hochberg *p* value = 0.243, Figure 9B). Furthermore, within the perirhinal cortex, the DRSP + EE High group had a positive correlation between GAD67 expression and WMC errors on Trial 4 during the Early Acquisition Phase (*R*^2^ = 0.72, *p* < 0.001, Benjamini-Hochberg *p* value = 0.243, Figure 9C), whereby higher perirhinal cortex GAD67 expression was associated with more WMC errors on the maximum working memory load trial. Interestingly, the DRSP + EE-High group had a negative correlation between GAD65 expression and WMI errors on Trial 3 during the Early Acquisition Phase, with greater GAD65 expression levels in the perirhinal cortex were associated with fewer WMI errors on the moderate working memory load trial (*R*^2^ = 0.72, *p* < 0.001, Benjamini-Hochberg *p* value = 0.243, Figure 9D).

## Discussion

Drospirenone’s unique pharmacological properties (Fuhrmann et al., 1996; Schindler et al., 2003; Kuhl, 2005; Bitzer and Paoletti, 2009) and its continued popularity in the clinic (The Medical Letter on Drugs Therapeutics, 2020) merited investigation into its impact on the brain and behavior. Collectively, we showed dose-dependent cognitive benefits of drospirenone in young Ovx rats, which were modified by the addition of the synthetic estrogen EE. Brain assessments indicated that while drospirenone alone did not impact GAD65, GAD67, or IGF1-R expression within the parameters tested, EE modified GAD65 and GAD67 expression, providing a putative mechanism through which this synthetic estrogen impacts cognition, in a similar fashion to the interplay between endogenous E2 and GABAergic activity (Moura and Petersen, 2010). Relationships between protein expression and working memory performance were evident, although they were dependent upon WRAM phase, brain region, and hormone treatment.

### Water Radial-Arm Maze: Spatial Working Memory

In Experiment 1, the Medium dose of drospirenone, which most closely models the typical ratio of drospirenone to EE used in combined oral contraceptive formulations, had beneficial effects on spatial working memory when memory load was taxed compared to Ovx-Vehicle-treated rats. The Low and High doses of drospirenone showed working memory benefits during the Early Acquisition Phase, but only the Medium drospirenone dose continued to elicit benefits for spatial working memory when working memory was maximally taxed in the Asymptotic Phase. A 6-h delay on the WRAM significantly impaired working memory performance for Vehicle and DRSP-Low treated groups, but not the DRSP-Medium and DRSP-High groups, on the post-delay trial, suggesting the potential of a dose-dependent effect for delayed memory retention. Drospirenone’s dose-dependent effects may follow a *U*-shaped pattern, where too little or too high of a dose negatively impacted performance, while the medium dose provided an optimal range to exert meaningful positive effects on memory performance. Prior research in women has demonstrated similar GABA-mediated *U*-shaped effects of allopregnanolone on mood (Andréen et al., 2009; Bäckström et al., 2015); animal models have also shown *U*-shape effects of reproductive hormones on cognition, including rapid effects mediated by membrane-bound receptors (Acosta et al., 2009; Foster, 2012).

In Experiment 2, drospirenone was administered alone and in combination with two doses of EE that reflected commonly prescribed doses in combined oral contraceptives. We replicated the finding from Experiment 1 that drospirenone alone enhanced spatial working memory when memory load was taxed compared to Ovx rats without hormone treatment during the Early Acquisition Phase. Drospirenone-treated rats also had enhanced performance compared to both combinations of DRSP + EE in the Early Acquisition Phase, suggesting that concomitant EE administration attenuated the beneficial effects of drospirenone alone. These results support and extend prior findings from our laboratory with levonorgestrel, wherein this progestin was beneficial when administered alone, but resulted in spatial working memory impairments when administered in combination with E2 in middle-aged Ovx rats (Prakapenka et al., 2018). During Late Acquisition, EE-High treatment impaired working memory compared to DRSP + EE-High treatment, indicating a potential mnemonic benefit of combined hormone treatment in relation to EE-only treatment, at least at a high dose. However, Low-EE treated rats made fewer working memory errors than combined DRSP + Low-EE-treated rats during Late Acquisition, suggesting that working memory outcomes could be dependent on both dose and combination of hormones in this young adult Ovx model.

During the delayed memory retention evaluation, EE-Low, EE-High, and DRSP + EE-Low groups were impaired, but the Ovx-Vehicle group did not show a significant delay-induced impairment. Similar to the first experiment, both the DRSP group and the DRSP + EE-High group did not exhibit poorer performance following the delay. These findings point to a potential protective effect of drospirenone on delayed memory retention alone and in combination with EE-High treatment.

### Morris Water Maze: Spatial Reference Memory

In Experiment 1, the DRSP-Medium and DRSP-High doses showed benefits for RM performance compared to Ovx Vehicle-treated rats across all days of MM. This effect of the Medium drospirenone dose was not found in Experiment 2. In fact, there were no differences in performance for any comparison on the MM task in Experiment 2; thus, drospirenone may have a more consistent beneficial effect in the working memory domain. In both experiments, all groups spatially localized to the platform location by the end of testing.

### Open Field Task: Locomotor and Anxiety-Like Behavior

In Experiment 1, drospirenone administration alone at any dose did not impact locomotor or anxiety-like behavior. In Experiment 2, rats treated with a high dose of EE alone and in combination with drospirenone exhibited increased locomotor activity. Rats treated with a high dose of EE also had increased center distance and time, indicative of decreased anxiety-like behavior. These group differences may have been due, in part, to an overall change in locomotor activity induced by the high EE dose; as such, these findings should be interpreted with caution in the context of being solely related to anxiety-like alleviation by the high dose of EE alone and in combination with drospirenone, specifically. E2 levels are known to impact locomotor and anxiety-like behavior, and thus this finding is concordant with prior research in surgical and transitional menopause models (Blizard et al., 1975; Lund et al., 2005; Hiroi et al., 2006; Hiroi and Neumaier, 2006; McLaughlin et al., 2008; Koebele et al., 2021a). The investigation of effects of EE on anxiety assessments are currently limited, although dose-dependent beneficial effects of EE have been reported in ovary-intact rats (Simone et al., 2015). While drospirenone has beneficial impacts on PMDD symptoms and mood in the clinical setting (Paoletti et al., 2004; Foidart, 2005; Pearlstein et al., 2005; Yonkers et al., 2005; Fenton et al., 2007; Nappi et al., 2009; Archer et al., 2015; Hofmeister and Bodden, 2016), it is possible that the detrimental effect of Ovx on anxiety-like behavior (Blizard et al., 1975; Hiroi and Neumaier, 2006; Diz-Chaves et al., 2012; Hiroi et al., 2016) overrode any potential benefit of drospirenone alone in this assessment. Future studies should evaluate ovary-intact animals as well as implement additional tasks that measure more nuanced aspects of anxiety-like behavior to further investigate the role of drospirenone on specific symptoms of anxiety, mood, depression, and affect in a preclinical model.

### Peripheral Measures of Hormone Treatment

Drospirenone alone did not alter body weight, uterine weight, or induce vaginal cytology changes compared to vehicle treatment in Ovx rats in Experiment 1. In Experiment 2, all rats treated with EE, alone or in combination with drospirenone, weighed less than Vehicle-treated rats. In addition, rats treated with drospirenone weighed more than rats treated with a combination of drospirenone and EE at both doses. This was somewhat surprising, given the anti-mineralocorticoid receptor properties of drospirenone as it pertains to water retention and metabolic effects (Muhn et al., 1995; Fuhrmann et al., 1996; Sitruk-Ware, 2004, 2006; Foidart, 2005). However, it is important to note that all rats were surgically menopausal and did not have ovaries; therefore, it is possible that the detrimental metabolic effects of Ovx impacted body weight over and above any potential effect that drospirenone could have had on body weight. To this end, an evaluation of drospirenone administration on weight maintenance or gain in ovary-intact rats would be informative in the future. All rats treated with EE, alone and in combination with drospirenone, exhibited vaginal cytology indicative of vaginal epithelium stimulation. Furthermore, uterine weight was increased for all EE-treated rats at the end of the experiment, supporting the idea that EE stimulates uterine tissue growth, a finding we have previously reported (Mennenga et al., 2015a). The dose of drospirenone given in combination with EE treatments did not attenuate uterine weight at the end of the experiment, suggesting the dose given was insufficient to counter EE-induced uterine stimulation, an effect previously observed in intact rats, but not widely studied (Adeyanju and Olatunji, 2019). Different ratios of drospirenone to EE will be important to investigate in future studies.

### Brain Assessment and Correlations With Behavior

Drospirenone alone did not alter proteins of interest associated with spatial learning and memory, including GAD65, GAD67, and IGF1-R in the dorsal hippocampus, entorhinal cortex, or perirhinal cortex. The observed beneficial cognitive impact of drospirenone may be regulated by different neural systems, or there may be region-dependent effects of drospirenone in brain areas that we did not assess herein. Alternatively, the chronic nature of Ovx and daily hormone administration could have led to reorganizational processes in the systems of interest by the time brains were evaluated. Another possibility is that drospirenone-induced alterations are not evident at the level of protein expression for the selected markers, at least at the time point and hormone modulation experimental parameters assessed.

In Experiment 2, the EE-Low group had increased GAD65 and GAD67 protein expression in the perirhinal cortex compared to Ovx rats without hormone treatment. Although the perirhinal cortex is traditionally associated with visual recognition memory, Schulz-Klaus and colleagues reported a reduction in anxiety-like behaviors by temporarily inactivating the perirhinal cortex through intracranial infusions of the GABA_*A*_ receptor agonist muscimol (Schulz-Klaus et al., 2005). Furthermore, infusion of E2 into the perirhinal and entorhinal cortices, as well as systemic E2 administration, impaired performance on a delayed non-match-to-sample object recognition test, but enhanced novelty preference (Gervais et al., 2013, 2016), suggesting a broader role for the perirhinal and entorhinal cortices in complex cognitive tasks. Interestingly, the EE-Low group had enhanced working memory compared to the DRSP + EE-Low group, yet exhibited greater anxiety-like behaviors compared to the EE-High group in the OFT. Thus, the outcomes associated with changes in perirhinal cortex GAD expression following synthetic EE administration may be dose- and task- dependent, and should be further explored.

Within the perirhinal cortex, greater GAD67 and IGF1-R expression was correlated with increased WMC errors during the Early Acquisition Phase of the WRAM in DRSP + EE-High and DRSP + EE-Low groups, respectively. Although endogenous E2 has been associated with increased IGF1-R expression and long-term cognitive benefits (Witty et al., 2013), poorer performance associated with higher levels of IGF1-R suggests that either the synthetic E2 analog EE plays a unique role in IGF1-R expression, or that the addition of the synthetic progestin drospirenone alters the relationship between IGF1-R and enhanced cognition. Within the DRSP + EE-High group, greater GAD65 expression was associated with fewer WMI errors, indicating that the perirhinal cortex has a more complex role in spatial working memory than previously noted. In the entorhinal cortex, which is a gateway for information transfer between the hippocampus and other cortical regions in the context of learning (Kitamura et al., 2015), greater GAD65 expression was associated with fewer errors in the EE-High group. Just as endogenous E2 is a known regulator of GABAergic activity in the hippocampal complex (Wójtowicz and Mozrzymas, 2010), synthetic EE may also modulate aspects of the GABAergic system in the context of learning and memory in a similar fashion.

Preclinical research on synthetic ovarian hormones in the context of cognition and neurobiological correlates is generally limited; yet, findings from work on endogenous ovarian hormones have helped our understanding. For example, as EE impacted memory and GAD expression herein, E2 has also been shown to increase GAD mRNA levels in the hippocampus, regulate GABAergic activity, initiate IGF1-R signaling (Weiland, 1992; Murphy et al., 1998; Nakamura et al., 2004; Garcia-Segura et al., 2007), increase hippocampal IGF1-R expression (Witty et al., 2013), improve working memory, and decrease anxiety-like behavior following Ovx (Bimonte and Denenberg, 1999; Hiroi et al., 2006, 2016; Hiroi and Neumaier, 2006). Concomitant natural progesterone treatment has been shown to reverse the effect of E2 on hippocampal GAD mRNA expression (Weiland, 1992) and on neurotrophin expression (Bimonte-Nelson et al., 2004a), as well as attenuate E2’s cognitive benefits in surgical and transitional menopause models (Bimonte-Nelson et al., 2004b,^2006^; Koebele et al., 2021a). Yet, progesterone has acute beneficial neuroprotective effects following ischemic injury (Singh and Su, 2013), an outcome which has recently been extended to synthetic progestins including drospirenone acting through a GABA-mediated mechanism (El Amki et al., 2019). The timing of hormone treatment after surgical menopause may also be key to interpreting findings related to neurobiological correlates. For example, Nakamura and colleagues demonstrated that GAD65 immunoreactive cells in the hippocampus decreased 10 days after Ovx compared to three days after Ovx, and E2 increased GAD65 immunoreactive cells 10 days, but not three days, after Ovx (Nakamura et al., 2004). Treatment in the current experiment began 48 h after surgery; thus, GAD-mediated effects may not be evident in all groups as a result of immediate hormone treatment. In the current report, the synthetic estrogen EE modulated memory performance, anxiety-like behaviors, and GAD expression in unique ways compared to reports of endogenous analogs. Thus, while EE and drospirenone are molecularly similar to natural E2 (Kuhl, 2005; Sitruk-Ware and Nath, 2011) and progesterone (Muhn et al., 1995; Fuhrmann et al., 1996; Schindler et al., 2003; Kuhl, 2005), respectively, careful consideration must be given to the dose and treatment regimen if the goal is to mimic or improve the effects of endogenous hormones. In the future, it will be important to further investigate additional parameters to parse the distinct effects of synthetic vs natural hormones on outcomes of interest. It is important to note that all major neurotransmitter systems are impacted by ovarian hormone loss and treatment (Barth et al., 2015); thus, other proteins and cellular signaling cascades, as well as a broad range of brain regions involved in spatial working memory and attention (e.g., frontal cortex), should be investigated to establish a mechanism for the cognitive effects observed with drospirenone.

## Conclusion

A growing body of research supports the tenet that oral contraceptives impact cognition and alter brain function in task- and composition- dependent manners (e.g., Gogos, 2013; Egan and Gleason, 2012; Beltz et al., 2015, 2022; Porcu et al., 2019; Taylor et al., 2020; Ycaza Herrera et al., 2020; Gravelsins et al., 2021; Lewis et al., 2022; Menting-Henry et al., 2022). Overall, the current series of experiments found that the spironolactone-derived progestin drospirenone has beneficial effects for spatial working memory performance in a young adult Ovx rat model, and that the synthetic estrogen EE has variable effects on behavior that depend on dose and combination with drospirenone. Future investigations using an ovary-intact rat model with these combination therapies would be beneficial to understand how clinically-prescribed treatments impact cognitive function in a normally-cycling reproductive system, as the majority of women prescribed combined oral contraceptives have an intact uterus and ovaries. The primary endpoint for behavior in this series of experiments focused on cognitive domains involving working memory and anxiety-like behaviors, as these domains are known to be impacted by changes in endogenous sex steroid hormones. Future studies would benefit from an expansion of cognitive domains and related brain areas evaluated, particularly given the changes observed with EE in the perirhinal cortex herein, which has a crucial role in recognition memory that is modulated by E2 (Petrulis and Eichenbaum, 2003; Gervais et al., 2013, 2016). Moreover, further investigations into the mechanistic mediators of how different synthetic estrogens and progestins affect brain functions, including probing a greater breadth of protein markers, dendritic spine density, and cellular signaling, all of which are known to be impacted by E2, will likely yield new breakthroughs and pathways of insight for future clinical treatments (Nelson et al., 2014; Gervais et al., 2015; Smith et al., 2016; Batallán Burrowes et al., 2021). While drospirenone has promise for beneficial cognitive effects, the search continues for an estrogen-progestin combination therapy that results in beneficial, or even null, cognitive outcomes in addition to its contraceptive and non-contraceptive peripheral benefits.

## Data Availability Statement

The original contributions presented in the study are included in the article/supplementary material; further inquiries can be directed to the corresponding author.

## Ethics Statement

The animal study was reviewed and approved by the Arizona State University Institutional Animal Care and Use Committee.

## Author Contributions

SK: conceptualization, methodology, validation, formal analysis, investigation, data curation, writing—original draft, writing—reviewing and editing, visualization, supervision, and project administration. MP: conceptualization, methodology, validation, formal analysis, investigation, data curation, writing—original draft, writing—reviewing and editing, and project administration. JP, CB-L, SN-S, VP, IS, HB, SP, and CC: investigation, validation, and writing—review and editing. HB-N: conceptualization, methodology, validation, formal analysis, funding acquisition, resources, writing—original draft, writing—reviewing and editing, visualization, supervision, and project administration. All authors contributed to the article and approved the submitted version.

## Conflict of Interest

The authors declare that the research was conducted in the absence of any commercial or financial relationships that could be construed as a potential conflict of interest.

## Publisher’s Note

All claims expressed in this article are solely those of the authors and do not necessarily represent those of their affiliated organizations, or those of the publisher, the editors and the reviewers. Any product that may be evaluated in this article, or claim that may be made by its manufacturer, is not guaranteed or endorsed by the publisher.

## References

[B1] Acosta J. I. Mayer L. Talboom J. S. Tsang C. W. S. Smith C. J. Enders C. K. (2009). Transitional versus surgical menopause in a rodent model: etiology of ovarian hormone loss impacts memory and the acetylcholine system. *Endocrinology* 150 4248–4259. 10.1210/en.2008-1802 19470706 PMC2736080

[B2] Adeyanju O. A. Olatunji L. A. (2019). Drospirenone-containing oral contraceptives do not affect glucose regulation and circulating corticosterone. *J. Basic Clin. Physiol. Pharmacol.* 30 1–9. 10.1515/jbcpp-2018-0184 31469652

[B3] Andréen L. Nyberg S. Turkmen S. van Wingen G. Fernández G. Bäckström T. (2009). Sex steroid induced negative mood may be explained by the paradoxical effect mediated by GABAA modulators. *Psychoneuroendocrinology* 34 1121–1132. 10.1016/j.psyneuen.2009.02.003 19272715

[B4] Archer D. F. Ahrendt H.-J. Drouin D. (2015). Drospirenone-only oral contraceptive: results from a multicenter noncomparative trial of efficacy, safety and tolerability. *Contraception* 92 439–444. 10.1016/j.contraception.2015.07.014 26232513

[B5] Bäckström T. Bixo M. Strömberg J. (2015). GABAA receptor-modulating steroids in relation to women’s behavioral health. *Curr. Psychiatry Rep.* 17:92. 10.1007/s11920-015-0627-4 26396092

[B6] Bakeman R. (2005). Recommended effect size statistics for repeated measures designs. *Behav. Res. Methods* 37 379–384. 10.3758/BF03192707 16405133

[B7] Barha C. K. Galea L. A. M. (2010). Influence of different estrogens on neuroplasticity and cognition in the hippocampus. *Biochim. Biophys. Acta Gen. Subj.* 1800 1056–1067. 10.1016/j.bbagen.2010.01.006 20100545

[B8] Barth C. Villringer A. Sacher J. (2015). Sex hormones affect neurotransmitters and shape the adult female brain during hormonal transition periods. *Front. Neurosci.* 9:37. 10.3389/fnins.2015.00037 25750611 PMC4335177

[B9] Batallán Burrowes A. A. Sundarakrishnan A. Bouhour C. Chapman C. A. (2021). G protein-coupled estrogen receptor-1 enhances excitatory synaptic responses in the entorhinal cortex. *Hippocampus* 31 1191–1201. 10.1002/hipo.23383 34399010

[B10] Beltz A. M. Hampson E. Berenbaum S. A. (2015). Oral contraceptives and cognition: a role for ethinyl estradiol. *Horm. Behav.* 74 209–217. 10.1016/j.yhbeh.2015.06.012 26122296

[B11] Beltz A. M. Loviska A. M. Kelly D. P. Nielson M. G. (2022). The link between masculinity and spatial skills is moderated by the estrogenic and progestational activity of oral contraceptives. *Front. Behav. Neurosci.* 15:777911. 10.3389/fnbeh.2021.777911 35153692 PMC8828973

[B12] Benjamini Y. Hochberg Y. (1995). Controlling the false discovery rate: a practical and powerful approach to multiple testing. *R. Stat. Soc.* 57 289–300. 10.1111/j.2517-6161.1995.tb02031.x

[B13] Bimonte H. A. Denenberg V. H. (1999). Estradiol facilitates performance as working memory load increases. *Psychoneuroendocrinology* 24 161–173. 10.1016/S0306-4530(98)00068-7 10101725

[B14] Bimonte H. A. Hyde L. A. Hoplight B. J. Denenberg V. H. (2000). In two species, females exhibit superior working memory and inferior reference memory on the water radial-arm maze. *Physiol. Behav.* 70 311–317. 10.1016/s0031-9384(00)00259-6 11006429

[B15] Bimonte-Nelson H. A. Daniel J. M. Koebele S. V. (2015). “The Mazes,” in *The Maze Book: Theories, Practice, and Protocols for Testing Rodent Cognition*, ed. Bimonte-Nelson H. A. (New York, NY: Springer), 37–72. 10.1007/978-1-4939-2159-1_2

[B16] Bimonte-Nelson H. A. Francis K. R. Umphlet C. D. Granholm A. C. (2006). Progesterone reverses the spatial memory enhancements initiated by tonic and cyclic oestrogen therapy in middle-aged ovariectomized female rats. *Eur. J. Neurosci.* 24 229–242. 10.1111/j.1460-9568.2006.04867.x 16882019

[B17] Bimonte-Nelson H. A. Singleton R. S. Williams B. J. Granholm A.-C. E. (2004b). Ovarian hormones and cognition in the aged female rat: II. progesterone supplementation reverses the cognitive enhancing effects of ovariectomy. *Behav. Neurosci.* 118 707–714. 10.1037/0735-7044.118.4.707 15301598

[B18] Bimonte-Nelson H. A. Nelson M. Granholm A.-C. E. (2004a). Progesterone counteracts estrogen-induced increases in neurotrophins in the aged female rat brain. *Neuroreport* 15 2659–2663. 10.1097/00001756-200412030-00021 15570173

[B19] Bitzer J. Paoletti A. M. (2009). Added benefits and user satisfaction with a low-dose oral contraceptive containing drospirenone. *Clin. Drug Investig.* 29 73–78. 10.2165/0044011-200929020-00001 19133702

[B20] Blizard D. A. Lippman H. R. Chen J. J. (1975). Sex differences in open field behavior in the rat: the inductive and activational role of gonadal hormones. *Physiol. Behav.* 14 601–608. 10.1016/0031-9384(75)90188-2 1135316

[B21] Braden B. B. Andrews M. G. Acosta J. I. Mennenga S. E. Lavery C. Bimonte-Nelson H. A. (2017). A comparison of progestins within three classes: differential effects on learning and memory in the aging surgically menopausal rat. *Behav. Brain Res.* 322 258–268. 10.1016/j.bbr.2016.06.053 27368418 PMC5195920

[B22] Braden B. B. Garcia A. N. Mennenga S. E. Prokai L. Villa S. R. Acosta J. I. (2011). Cognitive-impairing effects of medroxyprogesterone acetate in the rat: independent and interactive effects across time. *Psychopharmacology* 218 405–418. 10.1007/s00213-011-2322-4 21562760 PMC3787203

[B23] Braden B. B. Kingston M. L. Whitton E. Lavery C. Tsang C. W. S. Bimonte-Nelson H. A. (2015). The GABA-A antagonist bicuculline attenuates progesterone-induced memory impairments in middle-aged ovariectomized rats. *Front. Aging Neurosci.* 7:149. 10.3389/fnagi.2015.00149 26321945 PMC4536389

[B24] Braden B. B. Talboom J. S. Crain I. D. Simard A. R. Lukas R. J. Prokai L. (2010). Medroxyprogesterone acetate impairs memory and alters the GABAergic system in aged surgically menopausal rats. *Neurobiol. Learn. Mem.* 93 444–453. 10.1016/j.nlm.2010.01.002 20074654 PMC3397202

[B25] Centers for Disease Control and Prevention (2019). *Key Statistics from the National Survey of Family Growth: Contraception.* Available online at: https://www.cdc.gov/nchs/nsfg/key_statistics/c.htm#contraception (accessed May 9, 2019).

[B26] Chen S. Kumar N. Mao Z. Sitruk-Ware R. Brinton R. D. (2018). Therapeutic progestin segesterone acetate promotes neurogenesis: implications for sustaining regeneration in female brain. *Menopause* 25 1138–1151. 10.1097/GME.0000000000001135 29846284 PMC7731586

[B27] Chesler E. J. Juraska J. M. (2000). Acute administration of estrogen and progesterone impairs the acquisition of the spatial morris water maze in ovariectomized rats. *Horm. Behav.* 38 234–242. 10.1006/hbeh.2000.1626 11104641

[B28] Daniels K. Abma J. C. (2018). Current contraceptive status Among Women Aged 15–49: United States, 2015-2017. *NCHS Data Brief.* 327, 1–8. 10.1080/23293691.2022.2054670 33151146

[B29] Davison S. L. Bell R. J. Robinson P. J. Jane F. Leech J. Maruff P. (2013). Continuous-combined oral estradiol/drospirenone has no detrimental effect on cognitive performance and improves estrogen deficiency symptoms in early postmenopausal women: a randomized placebo-controlled trial. *Menopause* 20 1020–1026. 10.1097/gme.0b013e318287474f 23591255

[B30] Dayal M. Barnhart K. (2001). Noncontraceptive benefits and therapeutic uses of the oral contraceptive pill. *Semin. Reprod. Med.* 19 295–303. 10.1055/s-2001-18637 11727171

[B31] Diz-Chaves Y. Kwiatkowska-Naqvi A. Von Hülst H. Pernía O. Carrero P. Garcia-Segura L. M. (2012). Behavioral effects of estradiol therapy in ovariectomized rats depend on the age when the treatment is initiated. *Exp. Gerontol.* 47 93–99. 10.1016/j.exger.2011.10.008 22075533

[B32] Egan K. R. Gleason C. E. (2012). Longer duration of hormonal contraceptive use predicts better cognitive outcomes later in life. *J. Womens Health* 21 1259–1266. 10.1089/jwh.2012.3522 22994984 PMC3518542

[B33] El Amki M. Binder N. Steffen R. Schneider H. Luft A. R. Weller M. (2019). Contraceptive drugs mitigate experimental stroke-induced brain injury. *Cardiovasc. Res.* 115 637–646. 10.1093/cvr/cvy248 30295757

[B34] Fenton C. Wellington K. Moen M. Robinson D. (2007). Drugs: Drospirenone/Ethinylestradiol 3mg/20 μg (24/4 Day Regimen): A Review of Its Use in Contraception. Premenstrual Dysphoric Disorder and Moderate Acne Vulgaris. *Drugs* 67:67120. 10.2165/00003495-200767120-00007 17683173

[B35] Foidart J. M. (2005). Added benefits of drospirenone for compliance. *Climacteric* 8 28–34. 10.1080/13697130500330309 16203653

[B36] Foster T. C. (2012). Role of estrogen receptor alpha and beta expression and signaling on cognitive function during aging. *Hippocampus* 22 656–669. 10.1002/hipo.20935 21538657 PMC3704216

[B37] Frankfurt M. Luine V. (2015). The evolving role of dendritic spines and memory: interaction(s) with estradiol. *Horm. Behav.* 74 28–36. 10.1016/j.yhbeh.2015.05.004 25993604 PMC4573337

[B38] Fuhrmann U. Krattenmacher R. Slater E. P. Fritzemeier K. H. (1996). The novel progestin drospirenone and its natural counterpart progesterone: biochemical profile and antiandrogenic protential. *Contraception* 7824 243–251. 10.1016/s0010-7824(96)00195-3 8922878

[B39] Gallo-Oller G. Ordoñez R. Dotor J. (2018). A new background subtraction method for Western blot densitometry band quantification through image analysis software. *J. Immunol. Methods* 457 1–5. 10.1016/j.jim.2018.03.004 29522776

[B40] Garcia-Segura L. M. Sanz A. Mendez P. (2007). Cross-talk between IGF-I and estradiol in the brain: focus on neuroprotection. *Neuroendocrinology* 84 275–279. 10.1159/000097485 17124377

[B41] Gervais N. J. Hamel L. M. Brake W. G. Mumby D. G. (2016). Intra-perirhinal cortex administration of estradiol, but not an ERβ agonist, modulates object-recognition memory in ovariectomized rats. *Neurobiol. Learn. Mem.* 133 89–99. 10.1016/j.nlm.2016.06.012 27321161

[B42] Gervais N. J. Jacob S. Brake W. G. Mumby D. G. (2013). Systemic and intra-rhinal-cortical 17-β estradiol administration modulate object-recognition memory in ovariectomized female rats. *Horm. Behav.* 64 642–652. 10.1016/j.yhbeh.2013.08.010 24012943

[B43] Gervais N. J. Mumby D. G. Brake W. G. (2015). Attenuation of dendritic spine density in the perirhinal cortex following 17β-Estradiol replacement in the rat. *Hippocampus* 25 1212–1216. 10.1002/hipo.22479 26104963

[B44] Gogos A. (2013). Natural and synthetic sex hormones: effects on higher-order cognitive function and prepulse inhibition. *Biol. Psychol.* 93 17–23. 10.1016/j.biopsycho.2013.02.001 23410760

[B45] Goldman J. Murr A. Cooper R. (2007). The rodent estrous cycle: characterization of vaginal cytology and its utility in toxicological studies. *Birth Defects Res. B* 80 84–97. 10.1002/bdrb.20106 17342777

[B46] Gravelsins L. Duncan K. Einstein G. (2021). Do oral contraceptives affect young women’s memory? Dopamine-dependent working memory is influenced by COMT genotype, but not time of pill ingestion. *PLoS One* 16:e0252807. 10.1371/journal.pone.0252807 34111174 PMC8192013

[B47] Hall K. S. Trussell J. (2012). Types of combined oral contraceptives used by U.S. women. *Contraception* 86 659–665. 10.1016/j.contraception.2012.05.017 22770787 PMC3469779

[B48] Harburger L. L. Bennett J. C. Frick K. M. (2007). Effects of estrogen and progesterone on spatial memory consolidation in aged females. *Neurobiol. Aging* 28 602–610. 10.1016/j.neurobiolaging.2006.02.019 16621169

[B49] Harburger L. L. Saadi A. Frick K. M. (2009). Dose-dependent effects of post-training estradiol plus progesterone treatment on object memory consolidation and hippocampal extracellular signal-regulated kinase activation in young ovariectomized mice. *Neuroscience* 160 6–12. 10.1016/j.neuroscience.2009.02.024 19223011 PMC2668744

[B50] Hiroi R. McDevitt R. A. Neumaier J. F. (2006). Estrogen selectively increases tryptophan hydroxylase-2 mRNA expression in distinct subregions of rat midbrain raphe nucleus: association between gene expression and anxiety behavior in the open field. *Biol. Psychiatry* 60 288–295. 10.1016/j.biopsych.2005.10.019 16458260

[B51] Hiroi R. Neumaier J. F. (2006). Differential effects of ovarian steroids on anxiety versus fear as measured by open field test and fear-potentiated startle. *Behav. Brain Res.* 166 93–100. 10.1016/j.bbr.2005.07.021 16154649

[B52] Hiroi R. Weyrich G. Koebele S. V. Mennenga S. E. Talboom J. S. Hewitt L. T. (2016). Benefits of hormone therapy estrogens depend on estrogen type: 17β-estradiol and conjugated equine estrogens have differential effects on cognitive, anxiety-like, and depressive-like behaviors and increase tryptophan hydroxylase-2 mRNA levels in dorsal. *Front. Neurosci.* 10:517. 10.3389/fnins.2016.00517 28008302 PMC5143618

[B53] Hofmeister S. Bodden S. (2016). Premenstrual syndrome and premenstrual dysphoric disorder. *Am. Fam. Physician* 94 236–240. 27479626

[B54] Ikhena D. E. Johnson J. V. (2012). What are the options for providing contraception to perimenopausal women? *Sex. Reprod. Menopause* 10 1–6.

[B55] Joh H. Searles R. V. Selmanoff M. Alkayed N. J. Koehler R. C. Hurn P. D. (2006). Estradiol alters only GAD67 mRNA levels in ischemic rat brain with no consequent effects on GABA. *J. Cereb. Blood Flow Metab.* 26 518–526. 10.1038/sj.jcbfm.9600211 16094313 PMC1410818

[B56] Kitamura T. MacDonald C. J. Tonegawa S. (2015). Entorhinal-hippocampal neuronal circuits bridge temporally discontiguous events. *Learn. Mem.* 22 438–443. 10.1101/lm.038687.115 26286654 PMC4561404

[B57] Koebele S. V. Bimonte-Nelson H. A. (2016). Modeling menopause: the utility of rodents in translational behavioral endocrinology research. *Maturitas* 87 5–17. 10.1016/j.maturitas.2016.01.015 27013283 PMC4829404

[B58] Koebele S. V. Hiroi R. Plumley Z. M. T. Melikian R. Prakapenka A. V. Patel S. (2021a). Clinically used hormone formulations differentially impact memory, anxiety-like, and depressive-like behaviors in a rat model of transitional menopause. *Front. Behav. Neurosci.* 15:696838. 10.3389/fnbeh.2021.696838 34366807 PMC8335488

[B59] Koebele S. V. Quihuis A. M. Lavery C. N. Plumley Z. M. T. Castaneda A. J. Bimonte-Nelson H. A. (2021b). Oestrogen treatment modulates the impact of cognitive experience and task complexity on memory in middle-aged surgically menopausal rats. *J. Neuroendocrinol.* 33:e13002. 10.1111/jne.13002 34378820 PMC9124643

[B60] Koebele S. V. Mennenga S. E. Hiroi R. Quihuis A. M. Hewitt L. T. Poisson M. L. (2017). Cognitive changes across the menopause transition: a longitudinal evaluation of the impact of age and ovarian status on spatial memory. *Horm. Behav.* 87 96–114. 10.1016/j.yhbeh.2016.10.010 27793768 PMC5479707

[B61] Koebele S. V. Palmer J. M. Hadder B. Melikian R. Fox C. Strouse I. M. (2019). Hysterectomy uniquely impacts spatial memory in a rat model: a role for the non-pregnant uterus in cognitive processes. *Endocrinology* 160 1–19. 10.1210/en.2018-00709 30535329 PMC6293088

[B62] Krattenmacher R. (2000). Drospirenone: pharmacology and pharmacokinetics of a unique progestogen. *Contraception* 62 29–38. 10.1016/S0010-7824(00)00133-5 11024226

[B63] Kuhl H. (2005). Pharmacology of estrogens and progestogens: influence of different routes of administration. *Climacteric* 8 3–63. 10.1080/13697130500148875 16112947

[B64] Larivée N. Suissa S. Eberg M. Joseph L. Eisenberg M. J. Abenhaim H. A. (2016). Drospirenone-containing combined oral contraceptives and the risk of arterial thrombosis: a population-based nested case-control study. *BJOG* 124 1672–1679. 10.1111/1471-0528.14358 27704723

[B65] Lewis C. A. Kimmig A.-C. S. Kroemer N. B. Pooseh S. Smolka M. N. Sacher J. (2022). No differences in value-based decision-making due to use of oral contraceptives. *Front. Endocrinol.* 13:817825. 10.3389/fendo.2022.817825 (accessed on March 11, 2022). 35528016 PMC9075610

[B66] Liu J. H. (2021). The role of progestogens in menopausal hormone therapy. *Clin. Obstet. Gynecol.* 64 772–783. 10.1097/GRF.0000000000000657 34593694

[B67] Lowry N. C. Pardon L. P. Yates M. A. Juraska J. M. (2010). Effects of long-term treatment with 17 β-estradiol and medroxyprogesterone acetate on water maze performance in middle aged female rats. *Horm. Behav.* 58 200–207. 10.1016/j.yhbeh.2010.03.018 20362580 PMC2879457

[B68] Lund T. D. Rovis T. Chung W. C. J. Handa R. J. (2005). Novel actions of estrogen receptor-β on anxiety-related behaviors. *Endocrinology* 146 797–807. 10.1210/en.2004-1158 15514081

[B69] McDonald J. H. (2014). *Handbook of Biological Statistics.* (Baltimore, MD: Sparky House Publishing), 254–260.

[B70] McLaughlin K. J. Bimonte-Nelson H. A. Neisewander J. L. Conrad C. D. (2008). Assessment of estradiol influence on spatial tasks and hippocampal CA1 spines: evidence that the duration of hormone deprivation after ovariectomy compromises 17β-estradiol effectiveness in altering CA1 spines. *Horm. Behav.* 54 386–395. 10.1016/j.yhbeh.2008.04.010 18579142 PMC2602955

[B71] Mennenga S. E. Gerson J. E. Koebele S. V. Kingston M. L. Tsang C. W. S. Engler-Chiurazzi E. B. (2015a). Understanding the cognitive impact of the contraceptive estrogen Ethinyl Estradiol: tonic and cyclic administration impairs memory, and performance correlates with basal forebrain cholinergic system integrity. *Psychoneuroendocrinology* 54 1–13. 10.1016/j.psyneuen.2015.01.002 25679306 PMC4433884

[B72] Mennenga S. E. Koebele S. V. Mousa A. A. Alderete T. J. Tsang C. W. S. Acosta J. I. (2015b). Pharmacological blockade of the aromatase enzyme, but not the androgen receptor, reverses androstenedione-induced cognitive impairments in young surgically menopausal rats. *Steroids* 99 16–25. 10.1016/j.steroids.2014.08.010 25159107 PMC4398574

[B73] Menting-Henry S. Hidalgo-Lopez E. Aichhorn M. Kronbichler M. Kerschbaum H. Pletzer B. (2022). Oral contraceptives modulate the relationship between resting brain activity, amygdala connectivity and emotion recognition – a resting state fMRI Study. *Front. Behav. Neurosci.* 16:775796. 10.3389/fnbeh.2022.775796 35368304 PMC8967165

[B74] Miranda P. Williams C. L. Einstein G. (1999). Granule cells in aging rats are sexually dimorphic in their response to estradiol. *J. Neurosci.* 19 3316–3325. 10.1523/JNEUROSCI.19-09-03316.1999 10212291 PMC6782259

[B75] Morris R. (2015). “The watermaze,” in *The Maze Book: Theories, Practice, and Protocols for Testing Rodent Cognition*, ed. Bimonte-Nelson H. A. (New York, NY: Springer Publishing Humana Press), 73–92. 10.1007/978-1-4939-2159-1_3

[B76] Morris R. Garrud P. Rawlins J. N. O’Keefe J. (1982). Place navigation impaired in rats with hippocampal lesions. *Nature* 297 681–683. 10.1038/297681a0 7088155

[B77] Moura P. J. Petersen S. L. (2010). Estradiol acts through nuclear-and membrane-initiated mechanisms to maintain a balance between GABAergic and glutamatergic signaling in the brain: implications for hormone replacement therapy. *Rev. Neurosci.* 21 363–380. 10.1515/RNS.2011.022 21280455

[B78] Muhn P. Krattenmacher R. Beier S. Elger W. Schillinger E. (1995). Drospirenone: a novel progestogen with antimineralocorticoid and antiandrogenic activity. Pharmacological characterization in animal models. *Contraception* 51 99–110. 10.1016/0010-7824(94)00015-o 7750297

[B79] Murphy D. D. Cole N. B. Greenberger V. Segal M. (1998). Estradiol increases dendritic spine density by reducing GABA neurotransmission in hippocampal neurons. *J. Neurosci.* 18 2550–2559. 10.1523/jneurosci.18-07-02550.1998 9502814 PMC6793090

[B80] Nakamura N. H. Rosell D. R. Akama K. T. McEwen B. S. (2004). Estrogen and ovariectomy regulate mRNA and protein of glutamic acid decarboxylases and cation-chloride cotransporters in the adult rat hippocampus. *Neuroendocrinology* 80 308–323. 10.1159/000083657 15677881

[B81] Nappi R. E. Albani F. Tonani S. Santamaria V. Pisani C. Terreno E. (2009). Psychosexual well-being in women using oral contraceptives containing drospirenone. *Funct. Neurol.* 24 71–75. 19775533

[B82] Nelson B. S. Springer R. C. Daniel J. M. (2014). Antagonism of brain insulin-like growth factor-1 receptors blocks estradiol effects on memory and levels of hippocampal synaptic proteins in ovariectomized rats. *Psychopharmacology* 231 899–907. 10.1007/s00213-013-3310-7 24146138 PMC3945205

[B83] Olejnik S. Algina J. (2003). Generalized eta and omega squared statistics: measures of effect size for some common research designs. *Psychol. Methods* 8 434–447. 10.1037/1082-989X.8.4.434 14664681

[B84] Paoletti A. M. Lello S. Fratta S. Orrù M. Ranuzzi F. Sogliano C. (2004). Psychological effect of the oral contraceptive formulation containing 3 mg of drospirenone plus 30 μg of ethinyl estradiol. *Fertil. Steril.* 81 645–651. 10.1016/j.fertnstert.2003.08.030 15037415

[B85] Pazol K. Northcutt K. Patisaul H. Wallen K. Wilson M. (2009). Progesterone and medroxyprogesterone acetate differentially regulate alpha-4 subunit expression of GABA-A receptors in the CA1 hippocampus of female rats. *Physiol. Behav.* 97 58–61. 10.1016/j.physbeh.2009.01.021 19394921 PMC2688658

[B86] Pearlstein T. Bachmann G. Zacur H. Yonkers K. (2005). Treatment of premenstrual dysphoric disorder with a new drospirenone-containing oral contraceptive formulation. *Contraception* 72 414–421. 10.1016/j.contraception.2005.08.021 16307962

[B87] Petrulis A. Eichenbaum H. (2003). The perirhinal-entorhinal cortex, but not the hippocampus, is critical for expression of individual recognition in the context of the Coolidge effect. *Neuroscience* 122 599–607. 10.1016/j.neuroscience.2003.08.009 14622903

[B88] Pinkerton J. V. Sánchez Aguirre F. Blake J. Cosman F. Hodis H. Hoffstetter S. (2017). The 2017 hormone therapy position statement of The North American Menopause Society. *Menopause* 24 1–26. 10.1097/GME.0000000000000921 28650869

[B89] Porcu P. Serra M. Concas A. (2019). The brain as a target of hormonal contraceptives: evidence from animal studies. *Front. Neuroendocrinol.* 55:100799. 10.1016/j.yfrne.2019.100799 31614151

[B90] Prakapenka A. V. Hiroi R. Quihuis A. M. Carson C. Patel S. Berns-Leone C. (2018). Contrasting effects of individual versus combined estrogen and progestogen regimens as working memory load increases in middle-aged ovariectomized rats: one plus one does not equal two. *Neurobiol. Aging* 64 1–14. 10.1016/j.neurobiolaging.2017.11.015 29316527 PMC5820186

[B91] Rivera R. Yacobson I. Grimes D. (1999). The mechanism of action of hormonal contraceptives and intrauterine contraceptive devices. *Am. J. Obstet. Gynecol.* 181 1263–1269. 10.1016/S0002-9378(99)70120-1 10561657

[B92] Rosario E. R. Ramsden M. Pike C. J. (2006). Progestins inhibit the neuroprotective effects of estrogen in rat hippocampus. *Brain Res.* 1099 206–210. 10.1016/j.brainres.2006.03.127 16793026

[B93] Schindler A. E. Campagnoli C. Druckmann R. Huber J. Pasqualini J. R. Schweppe K. W. (2003). Classification and pharmacology of progestins. *Maturitas* 46 7–16. 10.1016/j.maturitas.2003.09.014 14670641

[B94] Schulz-Klaus B. Fendt M. Schnitzler H. U. (2005). Temporary inactivation of the rostral perirhinal cortex induces an anxiolytic-like effect on the elevated plus-maze and on the yohimbine-enhanced startle response. *Behav. Brain Res.* 163 168–173. 10.1016/j.bbr.2005.04.022 16029901

[B95] Simone J. Bogue E. A. Bhatti D. L. Day L. E. Farr N. A. Grossman A. M. (2015). Ethinyl estradiol and levonorgestrel alter cognition and anxiety in rats concurrent with a decrease in tyrosine hydroxylase expression in the locus coeruleus and brain-derived neurotrophic factor expression in the hippocampus. *Psychoneuroendocrinology* 62 265–278. 10.1016/j.psyneuen.2015.08.015 26352480

[B96] Singh M. Su C. (2013). Progesterone-induced neuroprotection: factors that may predict therapeutic efficacy. *Brain Res.* 1514 98–106. 10.1016/j.brainres.2013.01.027 23340161 PMC3672388

[B97] Sitruk-Ware R. (2004). Pharmacological profile of progestins. *Maturitas* 47 277–283. 10.1016/j.maturitas.2004.01.001 15063480

[B98] Sitruk-Ware R. (2006). New progestagens for contraceptive use. *Hum. Reprod. Update* 12 169–178. 10.1093/humupd/dmi046 16291771

[B99] Sitruk-Ware R. Nath A. (2010). The use of newer progestins for contraception. *Contraception* 82 410–417. 10.1016/j.contraception.2010.04.004 20933114

[B100] Sitruk-Ware R. Nath A. (2011). Metabolic effects of contraceptive steroids. *Rev. Endocr. Metab. Disord.* 12 63–75. 10.1007/s11154-011-9182-4 21538049

[B101] Smith C. C. Smith L. A. Bredemann T. M. Mcmahon L. L. (2016). 17β estradiol recruits GluN2B-containing NMDARs and ERK during induction of long-term potentiation at temporoammonic-CA1 synapses. *Hippocampus* 26 110–117. 10.1002/hipo.22495 26190171 PMC6661254

[B102] Taylor C. M. Pritschet L. Jacobs E. G. (2020). The scientific body of knowledge – whose body does it serve? A spotlight on oral contraceptives and the brain. *Front. Neuroendocrinol.* 60:100874. 10.1016/j.yfrne.2020.100874 33002517 PMC7882021

[B103] The Medical Letter on Drugs Therapeutics (2020). Drospirenone (Slynd) - A New Progestin-Only Oral Contraceptive. *JAMA* 323 1963–1964. 10.1111/aogs.13688 32427303

[B104] Weiland N. G. (1992). Glutamic acid decarboxylase messenger ribonucleic acid is regulated by Estradiol and Progesterone in the Hippocampus. *Endocrinology* 131 2697–2702. 10.1210/endo.131.6.1446611 1446611

[B105] Witty C. F. Gardella L. Perez M. Daniel J. (2013). Short-term estradiol administration in aging ovariectomized rats provides lasting benefits for memory and the hippocampus: a role for insulin-like growth factor-1. *Endocrinology* 154 842–852. 10.1210/en.2012-1698 23264616

[B106] Wójtowicz T. Mozrzymas J. W. (2010). Estradiol and GABAergic transmission in the hippocampus. *Vitam. Horm.* 82 279–300. 10.1016/S0083-6729(10)82015-1 20472144

[B107] Woner V. E. Koebele S. V. Northup-Smith S. N. Willeman M. N. Barker C. Schatzki-Lumpkin A. (2019). “The cognitive effects of the highly selective progestin segesterone acetate in a rat model of surgical menopause. Program No. 586.16/O19,” in *Proceedings of the 2019 Neuroscience Meeting Planner* (Chicago, IL: Society for Neuroscience).

[B108] Woolley C. S. McEwen B. S. (1993). Roles of estradiol and progesterone in regulation of hippocampal dendritic spine density during the estrous cycle in the rat. *J. Comp. Neurol.* 336 293–306. 10.1002/cne.903360210 8245220

[B109] Woolley C. S. McEwen B. S. (1994). Estradiol regulates hippocampal dendritic spine density via an N-methyl-D-aspartate receptor-dependent mechanism. *J. Neurosci.* 14 7680–7687. 10.1523/JNEUROSCI.14-12-07680.1994 7996203 PMC6576901

[B110] Woolley C. S. Weiland N. G. McEwen B. S. Schwartzkroin P. A. (1997). Estradiol increases the sensitivity of hippocampal CA1 pyramidal cells to NMDA receptor-mediated synaptic input: correlation with dendritic spine density. *J. Neurosci.* 17 1848–1859. 10.1523/jneurosci.17-05-01848.1997 9030643 PMC6573364

[B111] Ycaza Herrera A. Velasco R. Faude S. White J. D. Opitz P. C. Huang R. (2020). Brain activity during a post-stress working memory task differs between the hormone-present and hormone-absent phase of hormonal contraception. *Neurobiol. Stress* 13:100248. 10.1016/j.ynstr.2020.100248 33344703 PMC7739035

[B112] Yonkers K. Brown C. Pearlstein T. Foegh M. Sampson-Landers C. Rapkin A. (2005). Efficacy of a new low-dose oral contraceptive with drospirenone in premenstrual dysphoric disorder. *Obs. Gynecol.* 106 492–501. 10.1097/01.AOG 16135578

